# Nicotine Modifies Corticostriatal Plasticity and Amphetamine Rewarding Behaviors in Mice^1^,^2^,^3^

**DOI:** 10.1523/ENEURO.0095-15.2015

**Published:** 2016-02-02

**Authors:** Granville P. Storey, Gabriel Gonzalez-Fernandez, Ian J. Bamford, Matthew Hur, Jonathan W. McKinley, Lauren Heimbigner, Ani Minasyan, Wendy M. Walwyn, Nigel S. Bamford

**Affiliations:** 1 Department of Neurology, The Graduate Program in Neurobiology and Behavior, and Center on Human Development and Disability, University of Washington and Seattle Children’s Hospital, Seattle, Washington 98105; 2 Departments of Pediatrics and Psychology, the Graduate Program in Neurobiology and Behavior, and Center on Human Development and Disability, University of Washington and Seattle Children’s Hospital, Seattle, Washington 98105; 3 Department of Psychiatry and Biobehavioral Sciences, David Geffen School of Medicine, University of California Los Angeles, Los Angeles, California 90095; 4 Departments of Pediatrics and Neurology, Yale School of Medicine, New Haven, Connecticut 06520

**Keywords:** acetylcholine, addiction, amphetamine, self-administration, sensitization, striatum, nicotine

## Abstract

Corticostriatal signaling participates in sensitized responses to drugs of abuse, where short-term increases in dopamine availability provoke persistent, yet reversible, changes in glutamate release. Prior studies in mice show that amphetamine withdrawal promotes a chronic presynaptic depression in glutamate release, whereas an amphetamine challenge reverses this depression by potentiating corticostriatal activity in direct pathway medium spiny neurons. This synaptic plasticity promotes corticostriatal activity and locomotor sensitization through upstream changes in the activity of tonically active cholinergic interneurons (ChIs). We used a model of operant drug-taking behaviors, in which mice self-administered amphetamine through an in-dwelling catheter. Mice acquired amphetamine self-administration under fixed and increasing schedules of reinforcement. Following a period of abstinence, we determined whether nicotinic acetylcholine receptors modified drug-seeking behavior and associated alterations in ChI firing and corticostriatal activity. Mice responding to conditioned reinforcement showed reduced ChI and corticostriatal activity *ex vivo*, which paradoxically increased following an amphetamine challenge. Nicotine, in a concentration that increases Ca^2+^ influx and desensitizes α4β2*-type nicotinic receptors, reduced amphetamine-seeking behaviors following abstinence and amphetamine-induced locomotor sensitization. Nicotine blocked the depression of ChI firing and corticostriatal activity and the potentiating response to an amphetamine challenge. Together, these results demonstrate that nicotine reduces reward-associated behaviors following repeated amphetamine and modifies the changes in ChIs firing and corticostriatal activity. By returning glutamatergic activity in amphetamine self-administering mice to a more stable and normalized state, nicotine limits the depression of striatal activity in withdrawal and the increase in activity following abstinence and a subsequent drug challenge.

## Significance Statement

ChIs exert a strong influence on striatal function, and in combination with medium spiny neurons, are key mediators of cue and drug responses to psychostimulants, such as cocaine. As ChIs and corticostriatal terminals express nicotinic receptors, we used a new model of chronic psychostimulant use, amphetamine self-administration in mice, to examine the role of these receptors in modulating striatal responses to drug exposure. Nicotine activation of these receptors normalized many of the cellular and behavioral adaptations to chronic amphetamine, suggesting a novel target to offset the effects of chronic psychostimulants.

## Introduction

Psychostimulants, including amphetamine and cocaine, have a high potential for abuse as they acutely increase brain dopamine (DA) levels (Sulzer, 2011). Psychostimulants trigger long-lasting and parallel adaptations in striatal glutamate (Pierce et al., 1996; McFarland et al., 2003; Bamford et al., 2004a; Wang et al., 2013a) and acetylcholine (ACh; Bickerdike and Abercrombie, 1997, 1999), which contribute to the motor and neuropsychological symptoms of drug dependence (Kalivas, 2009).

Chronic psychostimulant use alters corticostriatal signaling (Kalivas 2009). Withdrawal induces a further adaptation by promoting a chronic presynaptic depression (CPD) in glutamate release from cortical projections within the dorsal striatum that disrupts normal DA filtering of corticostriatal activity (Bamford et al., 2008; Wang et al., 2013a). A drug challenge reverses CPD by increasing glutamate availability through a process called paradoxical presynaptic potentiation (PPP), when DA exerts an excitatory, rather than an inhibitory, effect on the corticostriatal pathway. By normalizing corticostriatal activity in withdrawal, PPP may provide a mechanism by which drug re-administration promotes physiological and behavioral stability, a feature supported in models of addiction (Ahmed and Koob, 2005).

The dorsal striatum is a key mediator of many of the reward-related behaviors. This structure is associated with the formation of compulsive drug-seeking habits (Yin et al., 2004; Everitt and Robbins, 2005, 2015), locomotor sensitization (Bamford et al., 2008), cue-dependent and reversal learning, cognitive flexibility (Quintana et al., 2012; Bertran-Gonzalez et al., 2013; Bradfield et al., 2013; Wang et al., 2013a), and sensorimotor conditioning (Aosaki et al., 1994). The changes in synaptic glutamate that occur during amphetamine withdrawal are analogous to measurements of extrasynaptic glutamate in the nucleus accumbens (NAc) that follow repeated cocaine (Pierce et al., 1996; McFarland et al., 2003). However, withdrawal from cocaine, but not amphetamine, modifies glutamatergic signaling in the ventral and dorsal striatum by upregulating postsynaptic AMPA and NMDA receptor surface expression (Nelson et al., 2009). This suggests that contributions from upstream circuitry differentially regulate corticostriatal activity following cocaine or amphetamine withdrawal.

Within the dorsal striatum, ChIs play a critical role in these behaviors and contribute toward CPD and PPP (Bamford et al., 2008; Wang et al., 2013a). Through an extensive arborization network, ChIs exert a strong influence on striatal information processing (Wilson et al., 1990). Although morphologically similar across striatal regions in the rodent brain (Gonzales and Smith, 2015), ChIs show distinct region-specific responses to cocaine (Benhamou et al., 2014). Such heterogeneity may be a result of restricted DA and glutamate cotransmission (Chuhma et al., 2004). For example, in the dorsolateral striatum DA induces a pause in ChI firing followed by a rebound in activity. DA increases ChI activity in the NAc shell, but reduces ChI firing in the NAc core. A single exposure to amphetamine increases this heterogeneity across regions (Chuhma et al., 2014), suggesting that local DA release induces region-specific striatal responses.

ACh acts at nicotinic and muscarinic receptors in the striatum to regulate DA (Zhang and Sulzer, 2004), glutamate (McGehee et al., 1995), and ACh availability in a region-specific manner (Zhou et al., 2001; Threlfell and Cragg, 2011). Nicotinic receptors, through their profile of activation and desensitization, modulate the probability of DA and glutamate release to affect psychostimulant-induced reward behaviors (Hansen and Mark, 2007; Bamford et al., 2008; Wang et al., 2013a; Li et al., 2014). We propose that the ChIs in the dorsolateral striatum, and their repertoire of nicotinic receptors, are a key mediator of the striatal response to amphetamine-induced DA and glutamatergic plasticity.

Using a model of contingent amphetamine exposure in mice, we have examined striatal adaptations induced by amphetamine. This model pairs single or multiple lever press with a drug infusion through an indwelling jugular catheter to generate a behavioral profile of amphetamine use. We then examined the electrophysiological profile of the effects of chronic amphetamine on the ChIs in the dorsolateral striatum, an important hub of habitual drug-seeking behaviors. Finally, we probed the involvement of the α4β2*- and α7*-type nicotinic acetylcholine receptor subtypes in blocking the presynaptic corticostriatal plasticity induced by amphetamine.

## Methods

### Animals

All animal procedures were performed in accordance with the authors’ university animal care committee’s regulations. Experiments started when male and female C57BL/6J mice (Jackson Laboratories) were 2–3 months old and completed by 6 months. Mice were housed together in a modified specific pathogen free vivarium with a 12 h light/dark cycle with *ad libitum* access to food and water. For some mice, a catheter (0.2 mm inner diameter, 0.4 mm outer diameter; Camcaths) was implanted in the right jugular vein; the mice were housed singly thereafter. The catheters were maintained by flushing daily with heparinized 0.9% saline (Thomsen and Caine, 2005; Cui et al., 2014) with Timentin (120 mg/ml) added for the first 7 d. We anesthetized mice with Beuthanasia (320 mg/kg, i.p.) or with ketamine (650 mg/kg, i.p.) and xylazine (44 mg/kg, i.p.) prior to euthanasia.

### Operant intravenous amphetamine self-administration

*Stage 1: sucrose pretraining*. After 2 d of a graded food restriction protocol with unrestricted access to water, all mice obtained and maintained a 10% reduction in body weight. A further 2 d of food restriction followed but with a 20% sucrose solution provided for 8 h each day. The mice habituated to the testing room containing the operant chambers during on these days (Med Associates). Over the following 5 d, they were trained to enter the magazine (2 d) and then press either of two operant levers (3 d) to obtain a 20% sucrose reward (delivered to the magazine) to a maximum of 30 reinforcers per session.

*Stage 2: amphetamine self-administration*. Food and water were provided *ad libitum* after the last sucrose training session and for the remainder of the experiment. Intravenous jugular catheters were implanted the day after the last sucrose session.

*A: acquisition*. Following 3 d of recovery, the mice were returned to the operant boxes for intravenous amphetamine (0.05 mg/kg) self-administration. The preferred lever during sucrose pretraining was designated as the active lever for amphetamine self-administration. A response on the designated active lever resulted in an amphetamine reinforcer (0.67 μl/g body weight) and a cue (house light extinguished for 30 s), during which no further drug could be obtained. A maximum of 50 reinforcers was allowed during the 2 h session and the session concluded when either parameter was obtained. Mice underwent a minimum of 10 d of acquisition training at a fixed ratio of 1 (FR1), during which one lever press resulted in one reinforcer. Mice with ≤20% variation and a minimum of 20 earned reinforcers over the preceding 3 d were advanced to the next stage, FR2, where two lever presses resulted in one reinforcer. Mice trained at this level for a minimum of 3 d until they obtained stable rates of reinforcers. Thereafter, they proceeded to FR5 for a minimum of 3 d, where five presses resulted in one reinforcer. Mice with ≤20% variation in earned reinforcers then progressed to the next phase. We tested catheter patency by infusing 1% propofol (20 μl) after FR1 and again after FR3 and FR5. We removed mice from the study if they failed to become transiently limp during the patency test.

*B: amphetamine challenge following incubation*. As the measured presynaptic adaptations are stable for 140 d following amphetamine injections (Bamford et al., 2008) and maintaining catheter patency in mice is technically challenging, a within-subject design was used to assess the effect of nicotine on amphetamine reward-seeking behaviors following 7 or 14 d of abstinence. Mice were randomly assigned to either an amphetamine group or an amphetamine with nicotine group, received a noncontingent amphetamine injection alone (1 mg/kg, i.p.) or amphetamine (1 mg/kg, i.p.) with nicotine (0.25 mg/kg, i.p.), and were then placed immediately in the same operant chamber as used during acquisition. Under an FR1 schedule of reinforcement, the number of active lever presses, inactive lever presses, and reinforcers (or cue presentations) were recorded for 30 min. After 7 d, these same reward-seeking behaviors were recorded in mice receiving the alternative treatment. Electrophysiology experiments were conducted within 30 d of the final FR5 session.

### Locomotor sensitization

Locomotor responses were measured using animal activity monitor cages (San Diego Instruments). Computer monitoring of four infrared beams, separated by 8.8 cm that cross the width of each chamber, recorded the number of times mice broke each beam. We measured locomotor activity in ambulations (2 consecutive beam interruptions) summated over 5 min intervals. On each test day, animals acclimated to individual activity chambers for 90 min to allow the animal to become accustomed to its behavioral cage before subsequent injections of either amphetamine (2 mg/kg, i.p.) or saline (10 µl/g, i.p.). Following each injection, the mice were placed back into their respective activity chamber and their ambulations were recorded for 90 min. To separate the effects of novelty from the pharmacological effects of the drug, animals were acclimated to the locomotor chambers and injected with saline on experiment days 1 and 2.

### Electrophysiology

Data were obtained from three to four mice per experiment using standard techniques to prepare slices for electrophysiology. Experiments in the dorsal striatum were performed using 250-μm-thick coronal sections containing the motor cortex and dorsal striatum, second to fourth frontal slice of caudate putamen [bregma, +1.54 to +0.62 mm]. To measure evoked EPSCs (eEPSCs) in medium spiny neurons (MSNs), experiments were performed on thicker 300 μm sagittal sections, obtained at an interaural distance range from 0.72 to 1.44 mm from midline. Brains were dissected and immediately submerged in ice-cold, carbogenated (95% O_2_, 5% CO_2_) artificial cerebrospinal fluid solution (ACSF) containing the following (in mm): 124 NaCl, 5 KCl, 26 NaHCO_3_, 1.25 NaH_2_PO_4_, 2 MgCl_2_, 2 CaCl_2_, and 10 glucose, pH 7.2–7.4, 290–310 mOsm. Slices (300 µm) were prepared on a vibratome then transferred to an incubating chamber containing carbogenated NMDG-recovery solution, containing the following (in mm): 100 *N*-methyl d-glucamine, 2.5 KCl, 1.2 NaH_2_PO_4_, 30 NaHCO_3_, 20 HEPES, 10 MgS0_4_, 0.5 CaCL_2_, and 25 glucose at 35°C, pH 7.3–7.4, 300–310 mOsm. After 5 min, the slices were transferred to carbogenated ACSF (vehicle; 3 ml/min) warmed to 35°C and performed electrophysiology experiments on upright Zeiss Axioskop FS or an Olympus BX51WI microscope.

Cell-attached recordings from ChIs and whole-cell recordings from MSNs in the dorsal striatum were obtained in voltage-clamp mode. MSNs were clamped at −70 mV. Cells were visualized in slices with the aid of infrared videomicroscopy coupled with differential interference contrast optics. ChIs were identified by size (∼18–25 μm) and repetitive firing in gap-free mode. Cell identification was confirmed by measuring passive and active membrane properties in whole-cell configuration (Wang et al., 2013a) and by labelling with 1% biocytin, according to published protocols (Joshi et al., 2009). The pipette internal solution contained the following (in mm): 119 KMeSO_4_, 1 MgCl_2_, 0.1 CaCl_2_, 10 HEPES, 1 EGTA, 12 phosphocreatine, 2 Na_2_ATP, and 0.7 Na_2_GTP, pH 7.2, 280–300 mOsm/L (Bennett and Wilson, 1999; Maurice et al., 2004). Currents were Bessel filtered at 2 kHz and were allowed to stabilize for 5 min after achieving a seal resistance >1 GΩ. Cells were removed from analysis if the seal resistance fell <1 GΩ or if the firing rate was <0.3 Hz or changed by >20% during the baseline (Bennett and Wilson, 1999).

Whole-cell recordings in acute striatal slices were used to measure miniature EPSCs (mEPSCs) in MSNs from saline-treated mice, amphetamine self-administering mice, and nonresponding mice. MSNs were identified by somatic size (∼8–12 µm) and typical passive basic membrane properties. There were no differences in passive membrane properties of MSNs from saline-treated, amphetamine self-administering, and nonresponding mice (membrane resistance: 118±19, 95±12, 107±20 MΩ; membrane capacitance: 80±8, 87±8, 80±14 pF; series resistance: 11±1, 11±2, 9±3MΩ; holding current: −150±16, −131±28, −159±39 pA; time constant: 0.9±0.1, 1±0.2, 0.8±0.3 ms, respectively).

The electrophysiological properties were monitored throughout the recording and cells were removed from further analysis if the series resistance changed by >20%. The patch pipette (4–7 MΩ) contained the following internal solution (in mm): 125 Cs-methanesulfonate, 3 KCl, 4 NaCl, 1 MgCl_2_, 5 MgATP, 5 EGTA, 8 HEPES, 1 Tris-GTP, 10 di-sodium phosphocreatine, 0.1 leupeptin, and 4 *N*-(2,6-dimethylphenylcarbamoylmethyl)triethylammonium bromide (QX-314), pH 7.2–7.3, 270–280 mOsm).

EPSCs were evoked by electrical stimulation of the deep cortical layers of the motor cortex using a twisted tungsten bipolar electrode at stimulation strengths adjusted to 1.5× threshold (0.6±0.2 mA). Paired current pulses of 200 μs in duration were delivered at 20 Hz every 30 s. eEPSC currents were Bessel filtered at 1 kHz. The paired-pulse ratio (PPR) was calculated by dividing the amplitude of the second pulse by that of the first pulse and then multiplying by 100. Cells demonstrating eEPSCs with variable latencies or prolonged durations suggesting polysynaptic responses were rejected. mEPSCs were Bessel filtered at 4 kHz and recorded in gap free mode. The Na^+^ channel antagonist tetrodotoxin (1 µM) was added to block spontaneous cortically-derived action potentials and isolate presynaptic terminal activity. Currents were digitized at 50 µs using Digidata 1440A data acquisition and pClamp10.2 software (Molecular Devices). Cell activity was analyzed with Clampfit (Molecular Devices) and Mini Analysis (Synaptosoft). Chemicals and ligands were obtained from Sigma-Aldrich.

### Spike analysis

The power spectra for recorded spike trains were calculated as the magnitude-squared of the Fourier transform (St-Pierre et al., 2014) using custom software designed in MATLAB (MathWorks). The nonuniform sampling rate of spike time data required a method for resampling at a uniform sampling rate. The time of each spike was rounded to the nearest thousandth to guarantee an equivalent time in a resampled train at 1000 Hz (Δ*t*=0.001 s) in which corresponding time instances were set to 1 in the new train and all others to 0. A sampling rate of 1000 Hz provided a high Nyquist frequency at 500 Hz, while limiting aliasing contribution for frequency bins <200 Hz (Bair et al., 1994).

Frequency spectra were computed by a Fast Fourier transform (FFT) according to Welch’s method using a division of 15 windows with 50% overlap (Table 1; Welch, 1967). A Hanning window was used to improve frequency accuracy and to reduce spectral leakage (Wickramarachi, 2003). Window overlap preserved spikes located near the tapered ends of the Hanning windows. The spectrum of each window was squared then normalized to its average power to preserve the relative peaks and dips at the lower frequencies of interest when averaging (Bair et al., 1994). We applied these processing operations at the Welch window level to improve spectral accuracy for each cell. Averaging a large number of windows decreased spectral variance at the expense of frequency resolution (Jokinen et al., 2000), which was compensated by greater window length of overlapping windows than that of non-overlapping windows. To reduce the direct current spike, we subtracted windows by their means to create a zero-mean spike train. Peak frequencies were the center of the bin with the maximum integral (Table 2). Bins started at each calculated frequency and extended out for a total of 10 points in each window. The peak frequencies of each cell were normalized to their corresponding power, which roughly approximated the peaks seen in the frequency spectrum. Frequency distributions were determined by creating a probability distribution of the 1/interspike interval (ISI) frequencies (Table 3). We averaged the distributions for each spike train in an experimental group to create an average distribution of that group and then calculated the peak frequencies using the same method used for the frequency spectra.

**Table 1.** MATLAB code for power spectrum computation

function [f, spec] = welchfft(Spikes, dt, numBins, pOverlap, Padding) %% WELCHFFT % % Calculates power spectrum by averaging Fast Fourier Transforms of % overlapping window divisions % % INPUTS: % Spikes (s): Times of spikes % dt: Sampling rate for new sampled train % numBins: Number of window divisions % pOverlap: Percent overlap between windows % Padding (optional): Number of zeros to zero-pad signal (increases frequency % resolution) % NOTE: Can change window type manually in the code (SEE LINE 69). % OUTPUTS: % f: Column vector of frequencies % spec: Column vector of normalized spectrum values % % EXAMPLE values used in this paper: % Spikes = (Spike times go here); % dt = 0.001; % numBins = 15; % pOverlap = 50; % Padding = 50; % [f, spec] = welchfft(Spikes, dt, numBins, pOverlap, Padding); % plot(f,spec); % % Reference: Welch (1967) The use of fast Fourier transform for the % estimation of power spectra: a method based on time averaging over % short, modified periodograms. IEEE Trans Audio Electroacoust 15:70–73. %% if nargin == 4 Padding = 0; end nTime = (Spikes(1):dt:Spikes(end)+dt); %New uniformly sampled times nTime(2,:) = 0; %Initialize spike train %Find equivalent times and set value at that time to 1 nTime(2,ismember(round(nTime(1,:).*(1/dt)).*dt, round(Spikes.*(1/dt)).*dt)) … = 1; %Spike train equals binary train (1,0) spikeTrain = nTime(2,:); N=length(spikeTrain); %Length of spike train fs = 1/dt; %Sampling rate of new spike train L = floor(N/(numBins-pOverlap/100×numBins+pOverlap/100)); %Number of points in %length of window Overlap_N = floor(L×pOverlap/100); %Number of points in each overlapping section %Set endpoints of the windows windowEndpoints = [linspace(1,1+(L-Overlap_N)×(numBins-1),numBins); … L:L-Overlap_N:length(spikeTrain)+(L-Overlap_N)/2]; %Fix error from floor due to percent input spikeTrain = [spikeTrain zeros(1,windowEndpoints(2,end)-length(spikeTrain))]; %Initalize windows windows = zeros(numBins, 1); %Compute fft for all windows for i = 1:numBins %Take current window from the spike train currTrain = [spikeTrain(windowEndpoints(1,i):windowEndpoints(2,i)) … zeros(1,Padding)]; %Can change window here by replacing line 71 with %options below: windowed = hanning(length(currTrain))'.*(currTrain); %windowed = blackman(length(currTrain))'.*(currTrain); %windowed = flattopwin(length(currTrain))'.*(currTrain); %windowed = hamming(length(currTrain))'.*(currTrain); %Take zero-mean, fft, and magnitude-squared windows(i,1:length(windowed)) = abs(fft(windowed-mean(windowed))).^2; %Normalize to the mean windows(i,:) = windows(i,:)./mean(windows(i,:)); end %Frequencies up to the nyquist f = linspace(0,fs/2,floor(length(windows)/2))'; %Average results of windows of corresponding spectra values spec = mean(windows(:,1:floor(length(windows)/2)))'; end

**Table 2.** MATLAB code for determining peak frequencies

function [peak,dist] = peakdetect(A, B, binsize) %% PEAKDETECT help % % Determines the peak value of a distribution. % % INPUTS: % A: *x* values of distribution. % B: distribution values. % binsize: Number of points to be included in a bin. % OUTPUTS: % peak: center of bin with the maximum distribution value. % dist: highest average distribution value for a bin. % % EXAMPLE: % A = (*x* values go here) % B = (distribution values go here) % binsize = 10 % [peak, dist] = peakdetect(A,B,binsize); % %% PEAKDETECT %The number of points to extend forward or backward %Step size of the distribution dx = A(2)-A(1); %Initialize integral array integral = zeros(length(B)-binsize+1,1); %Calculate the integrals for i = 1:length(B)- binsize + 1 %Take the sum of the *x* values multiplied by *y* values in the bin integral(i) = sum(*dx*×B(i:i+binsize-1)); end %Find the index of the center of the bin with the max integral j = find(integral==max(integral))+round(binsize/2); %Find the value of the max integral peak = A(j); %Return the average dist around the max distribution value dist = mean(B(j-round(binsize/2):j+round(binsize/2))); end

**Table 3.** MATLAB code for ISI frequency distribution

function [BinCenters, Dist] = ISIFreq(Spikes, Steps) %% ISIFREQ help % % Returns a histogram of 1/ISI frequencies. % % INPUTS: % Spikes: Times of spikes. % Steps (1/units of Spikes): Steps (boundaries) for the histogram % frequency count. % OUTPUTS: % BinCenters: Returns centers of the bins used for the histogram. % Dist: Probability distribution of the ISI Frequencies % % EXAMPLE values used in this paper % Spikes = (Spike times go here); % Steps = 0:0.005:10; % [BinCenters, Dist] = ISIFreq(Spikes, Steps); % figure % bar(BinCenters, Dist); % title 'ISI Frequency Distribution' % xlabel 'Frequencies' % ylabel 'Distribution' % %% ISIFREQ %Offset spikes by 1 and subtract for ISI's; ISIs = Spikes(2:end) - Spikes(1:end-1); %Calculate ISI Frequencies ISIFreqs = 1./ISIs; %Locates the bin centers of the steps BinCenters = [(Steps(1:end-1) + Steps(2:end))./2 Steps(end)]; %Run histogram count Counts = histc(ISIFreqs,Steps); %Convert to probability distribution Dist = Counts./sum(Counts); end

We used the Robust Gaussian Surprise (RGS; Ko et al., 2012) methods to analyze the burst and pause firing patterns in ChIs. The RGS method can accurately detect small bursts, small pauses, and strings of pause activity in individual ChIs (Tables 4 and 5). Unlike the Poisson Surprise method, which is limited by its assumption of a Poisson process (Legéndy and Salcman, 1985), and the Rank Surprise method, which does not accurately detect small bursts, the RGS method exhibits a robust adaptability to varying firing rates (Ko et al., 2012). It also provides many facets for statistical significance including both burst and pause information. A *p* value of 0.05 was used in the calculation of the central location of all ISI distributions and in the Bonferroni correction of the results. This method’s robustness is due to its normalization algorithm, which takes the base 10 logarithm of all the ISIs and locates the central location through the median and median absolute deviation of the distribution of a standard window length around each logarithmic ISI. Then, each central location was subtracted from its corresponding logarithmic ISI to form a normalized log_10_ ISI train. The median and median average deviation of the distribution of normalized ISIs were used to generate a cumulative Gaussian probability distribution, which was then used to determine surprise values for burst and pause seeds. Burst seeds were set as normalized log_10_ ISIs less than −2.58 times the median average deviation, whereas pause seeds were set as normalized log_10_ ISIs >+2.58 times the median average deviation. Normalized log_10_ ISIs (>0.05 s) in front or behind of the seeds were added if they decreased the likelihood, assuming a Gaussian distribution with mean and SD taken from the normalized log_10_ ISI distribution, of the occurrence of the burst or pause string according to the cumulative probability distribution. This method’s precise selection of spikes in each burst or pause string event by only including spikes that decrease the probability of the occurrence of the event, as well as the normalization that prevents the stringing of multiple small bursts in regions contribute to its accurate and robust performance. Patterns in bursts and pauses were determined using variable thresholds to connect these events (Table 6). All algorithms were implemented in MATLAB.

**Table 4.** MATLAB code for RGS burst and pause detection

function [Bursts, Pauses] = RGSDetect(Spikes, N_min, Steps, p, alpha)
%% RGSDETECT help
%
% Determines burst and pause interspike interval (ISI) thresholds and
% identifies burst and pause strings based on the Robust Gaussian Surprise
% (RGS) method.
%
% INPUTS:
% Spikes (s): Times of spikes in seconds.
% N_min: Minimum number of spikes to be considered a burst/pause
% string.
% Steps (log10(s)): Bin edges for histogram count (histc) of the
% log ISIs.
% p: Bottom and top p% used as outliers to calculate central
% location; keep p in range [0.05, 0.30] (default 0.05).
% alpha: Value used in Bonferroni correction; lower value of
% alpha to filter out false positives (default 0.05).
% NOTE: Requires MATLAB statistics toolbox.
%
% OUTPUTS:
% Bursts: Structure containing burst information.
% Bursts.BurstingSpikes (s): Column of times of all spike times
% included in a burst.
% Bursts.IBF (Hz): Column of intraburst frequency (IBF) of each
% burst.
% Bursts.NumSpikes: Column of number of spikes in each burst.
% Bursts.Windows (s): 2 Columns of start and end times of each
% burst.
% Pauses: Structure containing pause information.
% Pauses.AllSpikes (s): Start times of all ISIs that satisfy
% pause threshold.
% Pauses.AllLengths (s): Lengths of all ISIs that satisfy pause
% threshold.
% Pauses.PausingSpikes (s): Column of all spike times
% included in a pause string.
% Pauses.IPF (Hz): Column of intrapause frequency (IPF) of each
% pause string.
% Pauses.NumSpikes: Column of number of spikes in each pause
% string.
% Pauses.Windows (s): 2 Columns of start and end times of each
% pause string.
% NOTE: Rows of structure elements correspond to the same burst or
% pause.
% NOTE: Normalized Log ISI Distribution (NLISI) plot is used confirm
% central distribution is centered on 0. If distribution is not
% centered on 0, change p until it is. Use steps to adjust the
% *x*-axis.
%
% EXAMPLE values used in this paper:
% Spikes = (Spike times go here);
% N_min = 2;
% Steps = -3:0.005:1.5;
% *p* = 0.05;
% alpha = 0.05;
% [Bursts, Pauses] = RGSDetect(Spikes, N_min, Steps, p, alpha);
%
% REFERENCE: Ko et al. (2012)
% Detection of bursts and pauses in spike trains
% J Neurosci Methods 211:145–158
%
%%% NORMALIZED LOG ISI DISTRIBUTION
ISIs = Spikes(2:end)-Spikes(1:end-1); %Offset spikes by 1 and subtract
%for ISI's
LogISIs = log10(ISIs); %Take the log10 of ISI's
N = length(LogISIs); %N = number of ISIs
Q = max([20,floor(0.2×N)]); %Set window length as max of 20 and 20% of N
NLISITrain = zeros(1,length(LogISIs))'; %Initialize Normalized Log ISI Train
%Central Location of first 2×Q+1 ISIs
CentralLoc1 = ComputeCL(LogISIs(1:2×Q+1), Steps, p);
%Subtract Central Location (Normalize)
NLISITrain(1:Q) = LogISIs(1:Q) - CentralLoc1;
%Central Location of last 2×Q+1 ISIs
CentralLoc2 = ComputeCL(LogISIs(N-2×Q:N), Steps, p);
%Subtract Central Location (Normalize)
NLISITrain(N-Q+1:end) = LogISIs(N-Q+1:end) - CentralLoc2;
%For the middle portion
for i = Q+1:N-Q
%Compute central location for portion of index +/− Q and subtract
NLISITrain(i) = LogISIs(i) - ComputeCL(LogISIs(i-Q:i+Q), Steps, p);
end
%Get statistics of the NLISI train
med = median(NLISITrain);
pool_MAD = mad(NLISITrain);
CentralDistBounds = [med - pool_MAD×2.58 med + pool_MAD×2.58];
mu = median(NLISITrain);
sigma = mad(NLISITrain);
%Plot the NLISI Distribution
figure
hold on
%Run a smoothing pdf kernel.
NLISIpdf = pdf(fitdist(NLISITrain,'Kernel'), Steps);
NLISIpdf = NLISIpdf./sum(NLISIpdf);
plot(Steps, NLISIpdf,'g')
%Plot treshold lines
plot([CentralDistBounds(1) CentralDistBounds(1)], [0 max(NLISIpdf)], '–r')
plot([CentralDistBounds(2) CentralDistBounds(2)], [0 max(NLISIpdf)], '–b')
xlabel 'Normalized Log ISIs'
ylabel 'Probability'
title 'Normalized Log ISI Distribution'
%% BURST AND PAUSE STRING DETECTION
%Get index and ISI lengths of all ISIs that satisfy burst threshold
Burst_Thresh = CentralDistBounds(1);
BurstINDXS = 1:length(NLISITrain);
BurstINDXS(NLISITrain >= Burst_Thresh) = []; %Delete all indexes greater than
%the burst threshold
if ∼isempty(BurstINDXS)
%Matrix of all potential burst ISIs and their indexes
BurstsM = [NLISITrain(NLISITrain < Burst_Thresh)';BurstINDXS];
[∼,c] = size(BurstsM);
Burst_Seed = mat2cell(BurstsM,2,ones(1,c,1));
else
Burst_Seed = {};
Bursts = Burst_Seed;
end
%Loop through each potential burst ISI (Burst Seed)
for i = 1:length(Burst_Seed);
%Go forward and backward from the current burst until both conditions
%are unsatisfied
forward = 1;
backward = 1;
while forward ∥ backward
currBurst = cell2mat(Burst_Seed(i));
%Go forward 1 ISI
if forward
%Set current ISI as end of the current burst
currSpike = currBurst(:,end);
if currSpike(2) ∼= length(NLISITrain)
%q is number of spikes
[∼,q] = size(currBurst);
%P1 is probability burst will occur assuming Gaussian
%distribution with mean, mu×q, and std, sqrt(q)×sigma
P1 = normcdf(sum(currBurst(1,:)), mu×q, sqrt(q).×sigma);
testBurst = [currBurst [NLISITrain(currSpike(2)+1);…
currSpike(2)+1]];
%P2 is the same probability with the next ISI added to the
%burst
P2 = normcdf(sum(testBurst(1,:)), mu×(q+1), …
sqrt(q+1).×sigma);
%If the next ISI increased the probability of the burst
%occurring
if P2 >= P1
%Stop going forward
forward = 0;
else
%Otherwise, set the current burst seed to the tested
%burst
Burst_Seed{i} = testBurst;
end
else
%Stop going forward if at the end of the ISI train
forward = 0;
end
end
currBurst = cell2mat(Burst_Seed(i));
%Go backward 1 ISI
if backward
%Set current ISI as end of the current burst
currSpike = currBurst(:,1);
if currSpike(2) ∼= 1
%q is number of spikes
[∼,q] = size(currBurst);
%P1 is probability burst will occur assuming Gaussian
%distribution with mean, mu×q, and std, sqrt(q)×sigma
P1 = normcdf(sum(currBurst(1,:)), mu×q, sqrt(q).×sigma);
testBurst = [[NLISITrain(currSpike(2)-1);currSpike(2)-1] …
currBurst];
%P2 is the same probability with the next ISI added to the
%burst
P2 = normcdf(sum(testBurst(1,:)), mu*(q+1), …
sqrt(q+1).*sigma);
%If the next ISI increased the probability of the burst
%occurring
if P2 >= P1
%Stop going backward
backward = 0;
else
%Otherwise, set the current burst seed to the tested
%burst
Burst_Seed{i} = testBurst;
end
else
%Stop going backward if at the end of the ISI train
backward = 0;
end
end
end
end
if ∼isempty(Burst_Seed)
%Initialize BurstInfo
BurstInfo = zeros(length(Burst_Seed),3);
%Get start index of each burst
BurstInfo(:,1) = cellfun(@(x) x(2,1),Burst_Seed);
%Get end index of each burst
BurstInfo(:,2) = cellfun(@(x) x(2,end),Burst_Seed);
%Get P-value of each burst (probability of occurence assuming Gaussian
%distribution)
BurstInfo(:,3) = cellfun(@(x) normcdf(sum(x(1,:)), mu*length(x), …
sqrt(length(x)).×sigma),Burst_Seed);
%Filter out bursts less than minimum number of spikes specified by N_min
BurstInfo(BurstInfo(:,2)-BurstInfo(:,1)+2 < N_min,:) = [];
%Filter out overlapping bursts
no_overlap = 0;
i=1;
if ∼isempty(BurstInfo)
[r,∼] = size(BurstInfo);
if r ∼= 1
while ∼no_overlap
%If the indexes of the burst ISIs don't intersect
if isempty(intersect(BurstInfo(i,1):BurstInfo(i,2),…
BurstInfo(i+1,1):BurstInfo(i+1,2)))
%move to the next burst
i = i+1;
else
%If the intersect, choose the burst with the lower P
%value
if BurstInfo(i,3) <= BurstInfo(i+1,3)
BurstInfo(i+1,:) = [];
else
BurstInfo(i,:) = [];
end
end
%When the end is reached, stop
[r,∼] = size(BurstInfo);
if i == r
no_overlap = 1;
end
end
end
%r is the number of rows or the number of bursts
[r,∼] = size(BurstInfo);
Bursts.BurstingSpikes = [];
%for each burst, append the burst spikes
for i = 1:r
Bursts.BurstingSpikes = [Bursts.BurstingSpikes;…
Spikes(BurstInfo(i,1):BurstInfo(i,2)+1)];
end
end
%Bonferroni correction for false positives
KB = length(find(BurstInfo(:,3) < alpha));
BurstInfo(BurstInfo(:,3)×KB >= alpha,:) = [];
%Use the indexes in burst info to find the burst windows
Bursts.Windows = [Spikes(BurstInfo(:,1)) Spikes(BurstInfo(:,2)+1)];
%Use the indexes to find the number of spikes in each burst
Bursts.NumSpikes = BurstInfo(:,2) - BurstInfo(:,1) + 2;
%Use the number of spikes and windows to calculate the IBF
Bursts.IBF = Bursts.NumSpikes./(Bursts.Windows(:,2) - …
Bursts.Windows(:,1));
end
%Get index and ISI lengths of all NLISIs that satisfy pause threshold
Pause_Thresh = CentralDistBounds(2);
PauseINDXS = 1:length(NLISITrain);
%Delete all indexes less than the pause threshold
PauseINDXS(NLISITrain <= Pause_Thresh) = [];
if ∼isempty(PauseINDXS)
%Matrix of all potential pause string NLISIs and their indexes
PausesM = [NLISITrain(NLISITrain > Pause_Thresh)';PauseINDXS];
[∼,c] = size(PausesM);
Pause_Seed = mat2cell(PausesM,2,ones(1,c,1));
else
Pause_Seed = {};
Pauses = [];
end
%Loop through each potential pause string NLISI (Pause Seed)
for i = 1:length(Pause_Seed);
%Go forward and backward from the current pause string until both conditions
%are unsatisfied
forward = 1;
backward = 1;
while forward ∥ backward
currPause = cell2mat(Pause_Seed(i));
%Go forward 1 ISI
if forward
%Set current ISI as end of the current pause string
currPauseind = currPause(:,end);
if currPauseind(2) ∼= length(NLISITrain)
[∼,q] = size(currPause);
%P1 is probability pause string will occur assuming Gaussian
%distribution with mean, mu×q, and std, sqrt(q)×sigma
P1 = (1-normcdf(sum(currPause(1,:)), mu×q, sqrt(q).×sigma));
testPause = [currPause [NLISITrain(currPauseind(2)+1);…
currPauseind(2)+1]];
%P2 is the same probability with the next ISI added to the
%pause string
P2 = (1-normcdf(sum(testPause(1,:)), mu×(q+1), …
sqrt(q+1).×sigma));
%If the next ISI increased the probability of the pause
%string occurring
if P2 >= P1
%Stop going forward
forward = 0;
else
%Otherwise, set the current pause seed to the tested
%pause string
Pause_Seed{i} = testPause;
end
else
forward = 0;
end
end
currPause = cell2mat(Pause_Seed(i));
%Go backward 1 ISI
if backward
currPauseind = currPause(:,1);
if currPauseind(2) ∼= 1
[∼,q] = size(currPause);
%P1 is probability pause string will occur assuming Gaussian
%distribution with mean, mu×q, and std, sqrt(q)×sigma
P1 = (1-normcdf(sum(currPause(1,:)), mu×q, sqrt(q).×sigma));
testPause = [[NLISITrain(currPauseind(2)-1);…
currPauseind(2)-1] currPause];
%P2 is the same probability with the next ISI added to the
%pause string
P2 = (1-normcdf(sum(currPause(1,:)), mu×(q+1), …
sqrt(q+1).×sigma));
%If the next ISI increased the probability of the pause
%string occurring
if P2 >= P1
%Stop going forward
backward = 0;
else
%Otherwise, set the current pause seed to the tested
%pause string
Pause_Seed{i} = testPause;
end
else
backward = 0;
end
end
end
end
if ∼isempty(Pause_Seed)
%Initialize PauseInfo variable
PauseInfo = zeros(length(Pause_Seed),3);
%Starting indexes of pause strings
PauseInfo(:,1) = cellfun(@(x) x(2,1),Pause_Seed);
%Ending indexes of pause strings
PauseInfo(:,2) = cellfun(@(x) x(2,end),Pause_Seed);
%P-value of the pause strings
PauseInfo(:,3) = cellfun(@(x) (1-normcdf(sum(x(1,:)), mu×length(x), …
sqrt(length(x)).×sigma)),Pause_Seed);
%Minimum number of spikes filter
PauseInfo(PauseInfo(:,2)-PauseInfo(:,1) + 2 < N_min,:) = [];
%Filter out overlaps
no_overlap = 0;
i=1;
if ∼isempty(PauseInfo)
%r is number of current pause strings
[r,∼] = size(PauseInfo);
if r ∼= 1
while ∼no_overlap
%If the indexes of the burst ISIs don't intersect
if isempty(intersect(PauseInfo(i,1):PauseInfo(i,2),…
PauseInfo(i+1,1):PauseInfo(i+1,2)))
%Move to next pause string
i = i+1;
else
%Choose the pause string with lower P-value
if PauseInfo(i,3) <= PauseInfo(i+1,3)
PauseInfo(i+1,:) = [];
else
PauseInfo(i,:) = [];
end
end
%End if the last pause string is reached
[r,∼] = size(PauseInfo);
if i == r
no_overlap = 1;
end
end
end
end
%Use indexes to find start and end times
Pauses.Windows = [Spikes(PauseInfo(:,1)) Spikes(PauseInfo(:,2)+1)];
%Use indexes to determine number of spikes
Pauses.NumSpikes = PauseInfo(:,2) - PauseInfo(:,1) + 2;
%Use windows and numspikes to calculate IPF
Pauses.IPF = Pauses.NumSpikes./(Pauses.Windows(:,2) - …
Pauses.Windows(:,1));
Pauses.PausingSpikes = [];
%Add pausing spikes from each pause string to the pausingspikes element
[r,∼] = size(PauseInfo);
for i = 1:r
Pauses.PausingSpikes = [Pauses.PausingSpikes;…
Spikes(PauseInfo(i,1):PauseInfo(i,2)+1)];
end
end
%Bonferroni correction
KP = length(find(PauseInfo(:,3) < alpha));
PauseInfo(PauseInfo(:,3)×KP >= alpha,:) = [];
%Get all pauses using the pause indexes
Pauses.AllSpikes = Spikes(PauseINDXS);
%Get the lengths of all the pauses that satisfy the threshold
Pauses.AllLengths = ISIs(PauseINDXS);
end

**Table 5.** MATLAB code for computing central location used in RGS

function [CentralLocation] = ComputeCL(ISIs, Steps, p) %% COMPUTECL help % % Subroutine required for MATLAB code for RGS burst and pause detection % (Table 4). This subroutine computes the central location given an ISI train % using robust measures % of the median absolute difference (MAD), median, and central set. % % INPUTS: % ISIs (s): Lengths of ISIs in seconds. % Steps: Bin edges for histogram count (histc) of the ISIs. % p: Bottom and top p% used as outliers to calculate central % location; keep p in range [0.05, 0.30] (default 0.05). % NOTE: RGSDetect inputs log scale ISIs and Steps. % OUTPUTS: % CentralLocation: Central location of the ISI distribution % % REFERENCE: Ko et al. (2012) Detection of bursts and % pauses in spike trains. J Neurosci Methods 211:145–158 % %% COMPUTECL % %Locates the bin centers of the steps on a linear scale bincenters = [(Steps(1:end-1) + Steps(2:end))./2 Steps(end)]; %Histogram counts the ISIs using Steps ISIhist = histc (ISIs,Steps) '; %Converts to probability distribution normhist = ISIhist./sum(ISIhist); %Creates cumulative probability distribution cumprob = cumsum(normhist); %Calculates thresholds for bottom and top p quantiles [∼,burstquantid] = min(abs(cumprob-(p))); [∼,pausequantid] = min(abs(cumprob-(1-p))); burstquant = bincenters(burstquantid); pausequant = bincenters(pausequantid); %Caclulates E-Center as average of 2 thresholds E_Center = (burstquant + pausequant)/2; %Calculates central set using MAD CentralSetBoundaries = [E_Center - 1.64×mad(ISIs,1) E_Center + … 1.64×mad(ISIs,1)]; CentralSet = [bincenters(bincenters >=CentralSetBoundaries(1) & … bincenters <=CentralSetBoundaries(2)); normhist(bincenters … >=CentralSetBoundaries(1) & bincenters <=CentralSetBoundaries(2))]; %Calculates median of central set CentralDistCumProb = cumsum(CentralSet(2,:)./sum(CentralSet(2,:))); [∼,CentralLocationid] = min(abs(CentralDistCumProb - 0.5)); CentralLocation = CentralSet(1,CentralLocationid); end

**Table 6.** MATLAB code for finding burst-pause patterns using RGS output

function [BSPB, BDPB] = BurstPausePatternDetector(Bursts,Pauses,dt)
%% BURSTPAUSEPATTERNDETECTOR help
%
% Identifies patterns in bursting and pausing using outputs from
% RGSDetect.
%
% INPUTS:
% Bursts: Structure containing burst information obtained from RGSDetect.
% Pauses: Structure containing pause information obtained from RGSDetect.
% dt: Minimum threshold connecting burst and pause events. Argument can
% take single threshold or vector of thresholds.
%
% OUTPUTS:
% NOTE: Each structure element of both outputs have L cells containing
% NxM matrices, where L is the length of vector dt, N is the number
% of hits and M is the length of the pattern. Each element of the cell
% contains the results of its respective threshold in dt.
% NOTE: Thresholds dynamically attenuated so they do not fall past
% more than one event.
%
% BSPB: Structure containing patterns associated with string pauses.
% BSPB.BSPHits (s): Cell of 2 column matrices. The matrices
% contain string pauses falling within the threshold past bursts.
% Each row is one pattern where the first column contains burst
% start times, and the second column contains pause start times.
% BSPB.SPBHits (s): Cell of 2 column matrices. The matrices
% contain bursts falling within the threshold past string pauses.
% Each row is one pattern where the first column contains string
% pause start times, and the second column contains burst start times.
% BSPB.BSPBHits (s): Cell of 3 column matrices. The matrices
% contain bursts falling within the threshold past string pauses.
% Each row is one pattern where the first column contains string
% pause start times, and the second column contains burst start times.
% BDPB: Structure containing patterns associated with discrete
% pauses.
% BDPB.BDPHits (s): Cell of 2 column matrices. The matrices
% contain discrete pauses falling within the threshold past bursts.
% Each row is one pattern where the first column contains burst
% start times, and the second column contains pause start times.
% BDPB.DPBHits (s): Cell of 2 column matrices. The matrices
% contain bursts falling within the threshold past discrete pauses.
% Each row is one pattern where the first column contains
% discrete pause start times, and the second column contains
% burst start times.
% BDPB.BDPBHits (s): Cell of 3 column matrices. The matrices
% contain bursts falling within the threshold past discrete pauses.
% Each row is one pattern where the first column contains
% discrete pause start times, and the second column contains
% burst start times.
%
% EXAMPLE values used in this paper:
% Spikes = (Spike times go here);
% N_min = 2;
% Steps = -3:0.005:1.5;
% p = 0.05;
% alpha = 0.05;
% [Bursts, Pauses] = RGSDetect(Spikes, N_min, Steps, p, alpha);
% dt = [3 6 9 12 15 18 21];
% [BSPB, BDPB] = BurstPausePatternDetector(Bursts,Pauses,dt);
%
%% Initialization
BWindows = Bursts.Windows;
BurstStarts = BWindows(:,1);
BurstEnds = BWindows(:,2);
PWindows = Pauses.Windows;
PauseStarts = PWindows(:,1);
PauseEnds = PWindows(:,2);
DPStarts = Pauses.AllSpikes;
DPEnds = Pauses.AllSpikes + Pauses.AllLengths;
%% **Burst-String Pause-Burst** Search
%B-SP Sub-search
BSPHits = cell(length(dt),1);
for i = 1:length(dt)
%Add threshold to end of all burst events.
edges = [BurstEnds BurstEnds+dt(i)/60]';
edges = edges(:)';
%Fix ranges that overlap.
edges(diff(edges) <= 0) = edges(logical([0 diff(edges)<=0])) - 10^(-8);
%Histogram count the string pauses using ranges (edges).
[N,∼,bin] = histcounts(PauseStarts,edges);
%Take only the first string pause after each burst.
bin(logical([0;diff(bin) == 0]')) = 0;
%Use the results from even bins.
bin(mod(bin,2) == 0) = 0;
bin(bin ∼= 0) = 1;
N(2:2:end) = [];
%Assign results to BSPHits.
BSPHits(i) = {[BurstStarts(N∼=0) PauseStarts(logical(bin))]};
end
BSPB.BSPHits = BSPHits;
%SP-B Sub-search
SPBHits = cell(length(dt),1);
for i = 1:length(dt)
edges = [PauseEnds PauseEnds+dt(i)/60]';
edges = edges(:)';
edges(diff(edges) <= 0) = edges(logical([0 diff(edges)<=0])) - 10^(-8);
[N,∼,bin] = histcounts(BurstStarts,edges);
bin(logical([0;diff(bin) == 0]')) = 0;
bin(mod(bin,2) == 0) = 0;
bin(bin ∼= 0) = 1;
N(2:2:end) = [];
SPBHits(i) = {[PauseStarts(N∼=0) BurstStarts(logical(bin))]};
end
BSPB.SPBHits = SPBHits;
%B-SP-B Concatenation
BSPBHits = cell(length(dt),1);
for i = 1:length(dt)
%Obtain results from BSP and SPB
BSPi = BSPHits{i}; SPBi = SPBHits{i};
%Find common start times of string pauses.
[hits,ia,ib] = intersect(BSPi(:,2),SPBi(:,1));
%Concatenate the common BSP and SPB patterns into a 3 event pattern.
BSPBHits(i) = {[BSPi(ia,1) hits SPBi(ib,2)]};
end
BSPB.BSPBHits = BSPBHits;
%% **Burst-Discrete Pause-Burst** Search
%B-DP Sub-search
BDPHits = cell(length(dt),1);
for i = 1:length(dt)
edges = [BurstEnds BurstEnds+dt(i)/60]';
edges = edges(:)';
edges(diff(edges) <= 0) = edges(logical([0 diff(edges)<=0])) - 10^(-8);
[N,∼,bin] = histcounts(DPStarts,edges);
bin(logical([0;diff(bin) == 0]')) = 0;
bin(mod(bin,2) == 0) = 0;
bin(bin ∼= 0) = 1;
N(2:2:end) = [];
BDPHits(i) = {[BurstStarts(N∼=0) DPStarts(logical(bin))]};
end
BDPB.BDPHits = BDPHits;
%DP-B Sub-search
DPBHits = cell(length(dt),1);
for i = 1:length(dt)
edges = [DPEnds DPEnds+dt(i)/60]';
edges = edges(:)';
edges(diff(edges) <= 0) = edges(logical([0 diff(edges)<=0])) - 10^(-8);
[N,∼,bin] = histcounts(BurstStarts,edges);
bin(logical([0;diff(bin) == 0]')) = 0;
bin(mod(bin,2) == 0) = 0;
bin(bin ∼= 0) = 1;
N(2:2:end) = [];
DPBHits(i) = {[DPStarts(N∼=0) BurstStarts(logical(bin))]};
end
BDPB.DPBHits = DPBHits;
%B-DP-B Concatenation
BDPBHits = cell(length(dt),1);
for i = 1:length(dt)
BDPi = BDPHits{i}; DPBi = DPBHits{i};
[hits,ia,ib] = intersect(BDPi(:,2),DPBi(:,1));
BDPBHits(i) = {[BDPi(ia,1) hits DPBi(ib,2)]};
end
BDPB.BDPBHits = BDPBHits;
end

### Statistics

Values given in the text and in the figures are indicated as mean±SE. For the behavioral experiments, differences between groups across training days were analyzed using a linear mixed model, which can be used to maintain test validity by directly modeling unequal covariance across repeated measures (training day), which is intrinsic to a learning experiment, where responding starts off low and increases in variance (and mean) as days progress. These models included fixed effects for day and their interaction, along with an unstructured residual covariance matrix. For tests where equal variance and covariance could be assumed, as determined by Mauchley’s test of sphericity (because learning had already occurred, for example, stable responding in FR2 and progressive ratio data), two-way ANOVA (day and genotype) with repeated measures and factorial analysis were used. We compared locomotor ambulations using two-way ANOVA (day and genotype) with repeated measures. Differences in the electrophysiological tests were assessed with Student’s, paired, or Bonferroni *t* tests. Statistical analyses were performed with Statistica (StatSoft), or SPSS 21 (IBM) and differences were considered significant if *p*<0.05 (Table 7).

**Table 7.** Statistics

	**Dataset**	**Data structure**	**Type of test**	***p* value**
a	Fig. 1B. Active lever presses (saline-treated vs self-administering mice)	Normal distribution	Two-way ANOVA	<0.0001
b	Fig. 1D. Active vs total lever presses in saline-treated mice	Normal distribution	Two-way ANOVA	<0.0001
c	Fig. 1E. Drug reinforcers (saline-treated vs self-administering mice)	Normal distribution	Two-way ANOVA	<0.0001
d	Incubation effect of amphetamine challenge in self-administering mice	Normal distribution	paired *t* test	0.01
e	Fig. 1F. Active lever presses (self-administering mice without and with amphetamine and nicotine)	Normal distribution	paired *t* test	0.02
f	Fig. 1I. Cue reinforcers (self-administering mice without and with amphetamine and nicotine)	Normal distribution	paired *t* test	0.02
g	Fig. 1G. Number of inactive lever presses (self-administering mice treated with amphetamine vs self-administering mice treated with amphetamine and nicotine)	Normal distribution	paired *t* test	0.07
h	Fig. 1H. Percentage ratio of active/total lever presses (self-administering mice treated with amphetamine vs self-administering mice treated with amphetamine and nicotine)	Normal distribution	paired *t* test	0.15
i	Fig. 2A. Baseline firing frequency (saline-treated vs self-administering mice)	Normal distribution	Student’s *t* test	0.03
	Fig. 2B. Peak firing frequency power (saline-treated vs self-administering mice)	Normal distribution	Student’s *t* test	0.02
	Fig. 2D. Peak firing frequency distribution (saline-treated vs self-administering mice)	Normal distribution	Student’s *t* test	0.02
j	Fig. 2E. Firing frequency in saline-treated mice (vehicle vs amphetamine)	Normal distribution	paired *t* test	0.006
k	Fig. 2F. Firing frequency in self-administering mice (vehicle vs amphetamine)	Normal distribution	paired *t* test	0.04
p	Fig. 2E. Firing frequency in saline-treated mice (vehicle vs amphetamine with nicotine)	Normal distribution	paired *t* test	0.3
	Fig. 2E. Firing frequency in saline-treated mice (amphetamine vs amphetamine with nicotine)	Normal distribution	paired *t* test	0.04
q	Fig. 2F. Firing frequency in self-administering mice (vehicle vs amphetamine with nicotine)	Normal distribution	paired *t* test	0.3
	Fig. 2F. Firing frequency in self-administering mice (amphetamine vs amphetamine with nicotine)	Normal distribution	paired *t* test	0.08
	Fig. 2H. Peak firing frequency distribution in saline-treated mice (vehicle vs amphetamine)	Normal distribution	paired *t* test	0.03
	Fig. 2H. Peak firing frequency distribution in saline-treated mice (vehicle vs amphetamine with nicotine)	Normal distribution	paired *t* test	0.3
	Fig. 2H. Peak firing frequency distribution in saline-treated mice (amphetamine vs amphetamine with nicotine)	Normal distribution	paired t test	0.1
	Fig. 2I. Peak firing frequency distribution in self-administering mice (vehicle vs amphetamine)	Normal distribution	paired *t* test	0.008
	Fig. 2I. Peak firing frequency distribution in self-administering mice (vehicle vs amphetamine with nicotine)	Normal distribution	paired *t* test	0.09
	Fig. 2I. Peak firing frequency distribution in self-administering mice (amphetamine vs amphetamine with nicotine)	Normal distribution	paired *t* test	0.04
	Fig. 2J. Peak firing frequency distribution (saline-treated vs self-administering mice)	Normal distribution	Student’s *t* test	0.09
r	Fig. 3C. Discrete bursting (saline-treated vs self-administering mice)	Normal distribution	Student’s *t* test	0.03
	Fig. 3D. Intra-burst frequency (saline-treated vs self-administering mice)	Normal distribution	Student’s *t* test	0.7
s	Fig. 3D. Burst length in saline-treated mice (saline-treated vs self-administering mice)	Normal distribution	Student’s *t* test	0.03
t	Fig. 3D. Time bursting (saline-treated vs self-administering mice)	Normal distribution	Student’s *t* test	0.009
	Fig. 3E. Discrete pausing (saline-treated vs self-administering mice)	Normal distribution	Student’s *t* test	0.5
	Fig. 3F. Intra-pause frequency (saline-treated vs self-administering mice)	Normal distribution	Student’s *t* test	0.2
	Fig. 3F. Pause string length (saline-treated vs self-administering mice)	Normal distribution	Student’s *t* test	0.8
	Fig. 3F. Time pausing (saline-treated vs self-administering mice)	Normal distribution	Student’s *t* test	0.4
	Fig. 3G. Rate of burst and pause pattern occurrence (saline-treated vs self-administering mice)	Normal distribution	Student’s *t* test	0.2
	Fig. 3H. Rate of pause and burst pattern occurrence (saline-treated vs self-administering mice)	Normal distribution	Student’s *t* test	0.02
	Fig. 3I. Rate of burst-pause-burst pattern occurrence (saline-treated vs self-administering mice)	Normal distribution	Student’s *t* test	0.03
u	Fig. 4A. Discrete bursting in saline-treated mice (vehicle vs amphetamine)	Normal distribution	Paired *t* test	0.02
	Fig. 4A. Discrete bursting in saline-treated mice (vehicle vs amphetamine with nicotine)	Normal distribution	Paired *t* test	0.5
	Fig. 4A. Discrete bursting in saline-treated mice (amphetamine vs amphetamine with nicotine)	Normal distribution	Paired *t* test	0.2
	Fig. 4B. Intra-burst frequency in saline-treated mice (vehicle vs amphetamine)	Normal distribution	Paired *t* test	0.2
	Fig. 4B. Intra-burst frequency in saline-treated mice (vehicle vs amphetamine with nicotine)	Normal distribution	Paired *t* test	0.1
	Fig. 4B. Intra-burst frequency in saline-treated mice (amphetamine vs amphetamine with nicotine)	Normal distribution	Paired *t* test	0.3
	Fig. 4B. Burst length in saline-treated mice (vehicle vs amphetamine)	Normal distribution	Paired *t* test	0.1
	Fig. 4B. Burst length in saline-treated mice (amphetamine vs amphetamine with nicotine)	Normal distribution	Paired *t* test	0.2
	Fig. 4B. Burst length in saline-treated mice (vehicle vs amphetamine with nicotine)	Normal distribution	Paired *t* test	0.09
	Fig. 4B. Time spent bursting in saline-treated mice (vehicle vs amphetamine)	Normal distribution	Paired *t* test	0.4
	Fig. 4B. Time spent bursting in saline-treated mice (amphetamine vs amphetamine with nicotine)	Normal distribution	Paired *t* test	0.7
	Fig. 4B. Time spent bursting in saline-treated mice (vehicle vs amphetamine with nicotine)	Normal distribution	Paired *t* test	0.5
	Fig. 4C. Discrete pausing in saline-treated mice (vehicle vs amphetamine)	Normal distribution	Paired *t* test	0.2
	Fig. 4C. Discrete pausing in saline-treated mice (vehicle vs amphetamine with nicotine)	Normal distribution	Paired *t* test	0.3
	Fig. 4C. Discrete pausing in saline-treated mice (amphetamine vs amphetamine with nicotine)	Normal distribution	Paired *t* test	0.1
	Fig. 4D. Intra-pause frequency in saline-treated mice (vehicle vs amphetamine)	Normal distribution	Paired *t* test	0.4
	Fig. 4D. Intra-pause frequency in saline-treated mice (vehicle vs amphetamine with nicotine)	Normal distribution	Paired *t* test	0.6
	Fig. 4D. Intra-pause frequency in saline-treated mice (amphetamine vs amphetamine with nicotine)	Normal distribution	Paired *t* test	0.2
	Fig. 4D. Pause string length in saline-treated mice (vehicle vs amphetamine)	Normal distribution	Paired *t* test	0.06
	Fig. 4D. Pause string length in saline-treated mice (vehicle vs amphetamine with nicotine)	Normal distribution	Paired *t* test	0.2
	Fig. 4D. Pause string length in saline-treated mice (amphetamine vs amphetamine with nicotine)	Normal distribution	Paired *t* test	0.1
	Fig. 4D. Time spent pausing in saline-treated mice (vehicle vs amphetamine)	Normal distribution	Paired *t* test	0.2
	Fig. 4D. Time spent pausing in saline-treated mice (amphetamine vs amphetamine with nicotine)	Normal distribution	Paired *t* test	0.2
	Fig. 4D. Time spent pausing in saline-treated mice (vehicle vs amphetamine with nicotine)	Normal distribution	Paired *t* test	0.4
	Fig. 4E. Discrete bursting in self-administering mice (vehicle vs amphetamine)	Normal distribution	Paired *t* test	0.6
	Fig. 4E. Discrete bursting in self-administering mice (vehicle vs amphetamine with nicotine)	Normal distribution	Paired *t* test	0.6
	Fig. 4E. Discrete bursting in self-administering mice (amphetamine vs amphetamine with nicotine)	Normal distribution	Paired *t* test	0.7
	Fig. 4F. Intra-burst frequency in self-administering mice (vehicle vs amphetamine)	Normal distribution	Paired *t* test	0.1
	Fig. 4F. Intra-burst frequency in self-administering mice (amphetamine vs amphetamine with nicotine)	Normal distribution	Paired *t* test	0.07
	Fig. 4F. Intra-burst frequency in self-administering mice (vehicle vs amphetamine with nicotine)	Normal distribution	Paired *t* test	0.1
	Fig. 4F. Burst length in self-administering mice (vehicle vs amphetamine)	Normal distribution	Paired *t* test	0.1
	Fig. 4F. Burst length in self-administering mice (amphetamine vs amphetamine with nicotine)	Normal distribution	Paired *t* test	0. 08
	Fig. 4F. Burst length in self-administering mice (vehicle vs amphetamine with nicotine)	Normal distribution	Paired *t* test	0.007
	Fig. 4F. Time spent bursting in self-administering mice (vehicle vs amphetamine with nicotine)	Normal distribution	Paired *t* test	0.9
	Fig. 4F. Time spent bursting in self-administering mice (amphetamine vs amphetamine with nicotine)	Normal distribution	Paired *t* test	0.8
	Fig. 4F. Time spent bursting in self-administering mice (vehicle vs amphetamine with nicotine)	Normal distribution	Paired *t* test	0.1
v	Fig. 4G. Discrete pausing in self-administering mice (vehicle vs amphetamine)	Normal distribution	Paired *t* test	0.04
	Fig. 4G. Discrete pausing in self-administering mice (amphetamine vs amphetamine with nicotine)	Normal distribution	Paired *t* test	0.09
	Fig. 4G. Discrete pausing in self-administering mice (vehicle vs amphetamine with nicotine)	Normal distribution	Paired *t* test	0.5
w	Fig. 4H. Intra-pause frequency in self-administering mice (vehicle vs amphetamine)	Normal distribution	Paired *t* test	0.03
	Fig. 4H. Intra-pause frequency in self-administering mice (amphetamine vs amphetamine with nicotine)	Normal distribution	Paired *t* test	0.4
	Fig. 4H. Intra-pause frequency in self-administering mice (vehicle vs amphetamine with nicotine)	Normal distribution	Paired *t* test	0.1
	Fig. 4H. Pause string length in self-administering mice (vehicle vs amphetamine)	Normal distribution	Paired *t* test	0.1
	Fig. 4H. Pause string length in self-administering mice (amphetamine vs amphetamine with nicotine)	Normal distribution	Paired *t* test	0.1
	Fig. 4H. Pause string length in self-administering mice (vehicle vs amphetamine with nicotine)	Normal distribution	Paired *t* test	0.5
x	Fig. 4H. Time spent pausing in self-administering mice (vehicle vs amphetamine)	Normal distribution	Paired *t* test	0.04
	Fig. 4H. Time spent pausing in self-administering mice (amphetamine vs amphetamine with nicotine)	Normal distribution	Paired *t* test	0.08
	Fig. 4H. Time spent pausing in self-administering mice (vehicle vs amphetamine with nicotine)	Normal distribution	Paired *t* test	0.2
	Fig. 5A. Chl frequency (saline-treated vs self-administering mice)	Normal distribution	Student’s *t* test	0.03
y	Fig. 5A. Chl frequency in saline-treated mice (vehicle vs nicotine	Normal distribution	Paired *t* test	0.03
z	Fig. 5A. Chl frequency in self-administering mice (vehicle vs nicotine)	Normal distribution	Paired *t* test	0.04
	Fig. 5C. Peak frequency in saline-treated mice (vehicle vs nicotine)	Normal distribution	Paired *t* test	0.03
	Fig. 5D. Peak frequency in saline-treated mice (vehicle vs nicotine)	Normal distribution	Paired *t* test	0.04
	Fig. 5E. Peak frequency in saline-treated mice (vehicle vs nicotine)	Normal distribution	Student’s *t* test	0.02
aa	Fig. 5F. Discrete bursting in saline-treated mice (vehicle vs nicotine)	Normal distribution	Paired *t* test	0.02
	Fig. 5G. Intra-burst frequency in saline-treated mice (vehicle vs nicotine)	Normal distribution	Paired *t* test	0.5
	Fig. 5G. Burst length in saline-treated mice (vehicle vs nicotine)	Normal distribution	Paired *t* test	0.2
ab	Fig. 5G. Time bursting in saline-treated mice (vehicle vs nicotine)	Normal distribution	Paired *t* test	0.01
ac	Fig. 5H. Discrete pausing in saline-treated mice (vehicle vs nicotine)	Normal distribution	Paired *t* test	0.04
	Fig. 5I. Intra-pause frequency in saline-treated mice (vehicle vs nicotine)	Normal distribution	Paired *t* test	0.08
	Fig. 5I. Pause length in saline-treated mice (vehicle vs nicotine)	Normal distribution	Paired *t* test	0.7
	Fig. 5I. Time pausing in saline-treated mice (vehicle vs nicotine)	Normal distribution	Paired *t* test	0.1
ad	Fig. 5J. Discrete bursting in self-administering mice (vehicle vs nicotine)	Normal distribution	Paired *t* test	0.0008
	Fig. 5K. Intra-burst frequency in self-administering mice (vehicle vs nicotine)	Normal distribution	Paired *t* test	0.4
	Fig. 5K. Burst length in self-administering mice (vehicle vs nicotine)	Normal distribution	Paired *t* test	0.5
ae	Fig. 5K. Time bursting in self-administering mice (vehicle vs nicotine)	Normal distribution	Paired *t* test	0.01
af	Fig. 5L. Discrete pausing in self-administering mice (vehicle vs nicotine)	Normal distribution	Paired *t* test	0.02
ag	Fig. 5M. Intra-pause frequency in self-administering mice (vehicle vs nicotine)	Normal distribution	Paired *t* test	0.02
ah	Fig. 5M. Pause length in self-administering mice (vehicle vs nicotine)	Normal distribution	Paired *t* test	0.02
	Fig. 5M. Time pausing in saline-treated mice (vehicle vs nicotine)	Normal distribution	Paired *t* test	0.1
ai	Fig. 6A. mEPSC frequency (saline-treated mice vs self-administering mice)	Normal distribution	Student’s *t* test	0.04
aj	Fig. 6A. mEPSC frequency (saline-treated mice vs nonresponding mice)	Normal distribution	Student’s *t* test	0.003
ak	Fig. 6A. mEPSC frequency (self-administering mice vs nonresponding mice)	Normal distribution	Student’s *t* test	0.0003
	Fig. 6B. mEPSC frequency (saline-treated mice vs self-administering mice)	Normal distribution	Bonferroni *t* test	0.00003
	Fig. 6B. mEPSC frequency (saline-treated mice vs nonresponding mice)	Normal distribution	Bonferroni *t* test	0.002
	Fig. 6C. Peak frequency of mEPSCs (saline-treated mice vs self-administering mice)	Normal distribution	Student’s *t* test	0.009
	Fig. 6C. Peak frequency of mEPSCs (saline-treated mice vs nonresponding mice)	Normal distribution	Student’s *t* test	0.006
	Fig. 6C. Peak frequency of mEPSCs (self-administering mice vs nonresponding mice)	Normal distribution	Student’s *t* test	0.0008
al	Fig. 6D. mEPSC frequency in saline-treated mice (vehicle vs amphetamine)	Normal distribution	Paired *t* test	0.04
an	Fig. 6D. mEPSC frequency in saline-treated mice (vehicle vs amphetamine with nicotine)	Normal distribution	Paired *t* test	0.01
am	Fig. 6D. mEPSC frequency in saline-treated mice (amphetamine vs amphetamine with nicotine)	Normal distribution	Paired *t* test	0.04
	Fig. 6E. mEPSC frequency in saline-treated mice (vehicle vs amphetamine)	Normal distribution	Bonferroni *t* test	0.04
	Fig. 6E. mEPSC frequency in saline-treated mice (vehicle vs amphetamine with nicotine)	Normal distribution	Bonferroni *t* test	0.01
	Fig. 6E. mEPSC frequency in saline-treated mice (amphetamine vs amphetamine with nicotine)	Normal distribution	Bonferroni *t* test	0.09
	Fig. 6F. Peak frequency of mEPSCs in saline-treated mice (vehicle vs amphetamine)	Normal distribution	Paired *t* test	0.7
	Fig. 6F. Peak frequency of mEPSCs in saline-treated mice (vehicle vs amphetamine with nicotine)	Normal distribution	Paired *t* test	0.3
	Fig. 6F. Peak frequency of mEPSCs in saline-treated mice (amphetamine vs amphetamine with nicotine)	Normal distribution	Paired *t* test	0.2
ao	Fig. 6G. mEPSC frequency in self-administering mice (vehicle vs amphetamine)	Normal distribution	Paired *t* test	0.004
ap	Fig. 6G. mEPSC frequency in self-administering mice (amphetamine vs amphetamine with nicotine)	Normal distribution	Paired *t* test	0.006
	Fig. 6H. mEPSC frequency in self-administering mice (vehicle vs amphetamine)	Normal distribution	Bonferroni *t* test	0.04
	Fig. 6H. mEPSC frequency in self-administering mice (vehicle vs amphetamine with nicotine)	Normal distribution	Bonferroni *t* test	0.008
	Fig. 6H. mEPSC frequency in self-administering mice (amphetamine vs amphetamine with nicotine)	Normal distribution	Bonferroni *t* test	0.03
	Fig. 6I. Peak frequency of mEPSCs in self-administering mice (vehicle vs amphetamine)	Normal distribution	Paired *t* test	0.1
	Fig. 6I. Peak frequency of mEPSCs in self-administering mice (vehicle vs amphetamine with nicotine)	Normal distribution	Paired *t* test	0.09
	Fig. 6I. Peak frequency of mEPSCs in self-administering mice (amphetamine vs amphetamine with nicotine)	Normal distribution	Paired *t* test	0.04
	Fig. 6J. Peak frequency of mEPSCs (saline-treated mice vs self-administering mice with nicotine)	Normal distribution	Student’s *t* test	0.3
aq	Fig. 6K. mEPSC frequency in nonresponding mice (vehicle vs amphetamine)	Normal distribution	Paired *t* test	0.003
ar	Fig. 6K. mEPSC frequency in nonresponding mice (amphetamine vs amphetamine with nicotine)	Normal distribution	Paired *t* test	0.02
	Fig. 6L. mEPSC frequency in nonresponding mice (vehicle vs amphetamine)	Normal distribution	Bonferroni *t* test	0.003
	Fig. 6L. mEPSC frequency in nonresponding mice (vehicle vs amphetamine with nicotine)	Normal distribution	Bonferroni *t* test	0.0002
	Fig. 6L. mEPSC frequency in nonresponding mice (amphetamine vs amphetamine with nicotine)	Normal distribution	Bonferroni *t* test	0.03
	Fig. 6M. Peak frequency of mEPSCs in nonresponding mice (vehicle vs amphetamine)	Normal distribution	Paired *t* test	0.02
	Fig. 6M. Peak frequency of mEPSCs in nonresponding mice (vehicle vs amphetamine with nicotine)	Normal distribution	Paired *t* test	0.02
	Fig. 6M. Peak frequency of mEPSCs in nonresponding mice (amphetamine vs amphetamine with nicotine)	Normal distribution	Paired *t* test	0.2
as	Fig. 7A. mEPSC frequency in saline-treated mice (vehicle vs nicotine)	Normal distribution	Paired *t* test	0.04
	Fig. 7B. mEPSC frequency in saline-treated mice (vehicle vs nicotine)	Normal distribution	Bonferroni *t* test	0.04
	Fig. 7C. Peak frequency of mEPSCs in saline-treated mice (vehicle vs amphetamine)	Normal distribution	Paired *t* test	0.7
at	Fig. 7D. mEPSC frequency in self-administering mice (vehicle vs nicotine)	Normal distribution	Paired *t* test	0.03
	Fig. 7E. mEPSC frequency in self-administering mice (vehicle vs nicotine)	Normal distribution	Bonferroni *t* test	0.04
	Fig. 7F. Peak frequency of mEPSCs in self-administering mice (vehicle vs amphetamine)	Normal distribution	Paired *t* test	0.1
	Fig. 7G. mEPSC frequency in saline-treated mice (vehicle vs nicotine)	Normal distribution	Paired *t* test	0.04
au	Fig. 7G. mEPSC frequency (saline-treated mice vs self-administering mice)	Normal distribution	Student’s *t* test	0.002
av	Fig. 7G. mEPSC frequency in self-administering mice (vehicle vs nicotine)	Normal distribution	Paired *t* test	0.02
	Fig. 7H. mEPSC frequency (saline-treated mice vs self-administering mice with nicotine)	Normal distribution	Student’s *t* test	0.2
aw	Fig. 7I. mEPSC frequency in saline-treated mice (vehicle vs nicotine with DHβE)	Normal distribution	Paired *t* test	0.04
	Fig. 7J. mEPSC frequency in saline-treated mice (vehicle vs nicotine with DHβE)	Normal distribution	Bonferroni *t* test	0.03
ax	Fig. 7K. mEPSC frequency in saline-treated mice (vehicle vs nicotine with MLA)	Normal distribution	Paired *t* test	0.04
	Fig. 7L. mEPSC frequency in saline-treated mice (vehicle vs nicotine with MLA)	Normal distribution	Bonferroni *t* test	0.009
ay	Fig. 8B. Ambulations of saline-treated mice vs amphetamine-treated mice	Normal distribution	rm ANOVA	0.0001
az	Fig. 8C. Ambulations of saline-treated mice vs amphetamine-treated mice	Normal distribution	2-way rm-ANOVA	0.0001
ba	Fig. 8D. Ambulations of saline-treated mice (low-dose vs high-dose nicotine)	Normal distribution	2-way rm-ANOVA	0.9
bb	Fig. 8E. Ambulations of amphetamine-treated mice (amphetamine vs amphetamine with low-dose nicotine)	Normal distribution	2-way rm-ANOVA	1
bc	Fig. 8E. Ambulations of amphetamine-treated mice (amphetamine vs amphetamine with high-dose nicotine)	Normal distribution	2-way rm-ANOVA	0.01
bd	Fig. 8F. Ambulations of saline-treated mice (amphetamine vs amphetamine with high-dose nicotine)	Normal distribution	2-way rm-ANOVA	0.9
be	Fig. 8G. Ambulations of saline-treated mice challenged with nicotine vs amphetamine-treated mice challenged with nicotine	Normal distribution	2-way rm-ANOVA	0.9
bf	Fig. 8H. eEPSC amplitude in saline-treated mice (vehicle vs nicotine)	Normal distribution	Paired *t* test	0.02
bg	Fig. 8H. PPR in saline-treated mice (vehicle vs nicotine)	Normal distribution	Paired *t* test	0.009
bh	Fig. 8I. eEPSC amplitude in amphetamine-treated mice (vehicle vs nicotine)	Normal distribution	Paired *t* test	0.0008
bi	Fig. 8I. PPR in amphetamine-treated mice (vehicle vs nicotine)	Normal distribution	Paired *t* test	0.013
bj	Fig. 8H,I. eEPSC amplitude (saline-treated mice vs amphetamine-treated mice)	Normal distribution	Student’s *t* test	0.02
bk	Fig. 8H,I. eEPSC amplitude (saline-treated mice with nicotine vs amphetamine-treated mice with nicotine)	Normal distribution	Student’s *t* test	0.2

## Results

### Mice acquire stable intravenous amphetamine self-administration

**Figure 1.**
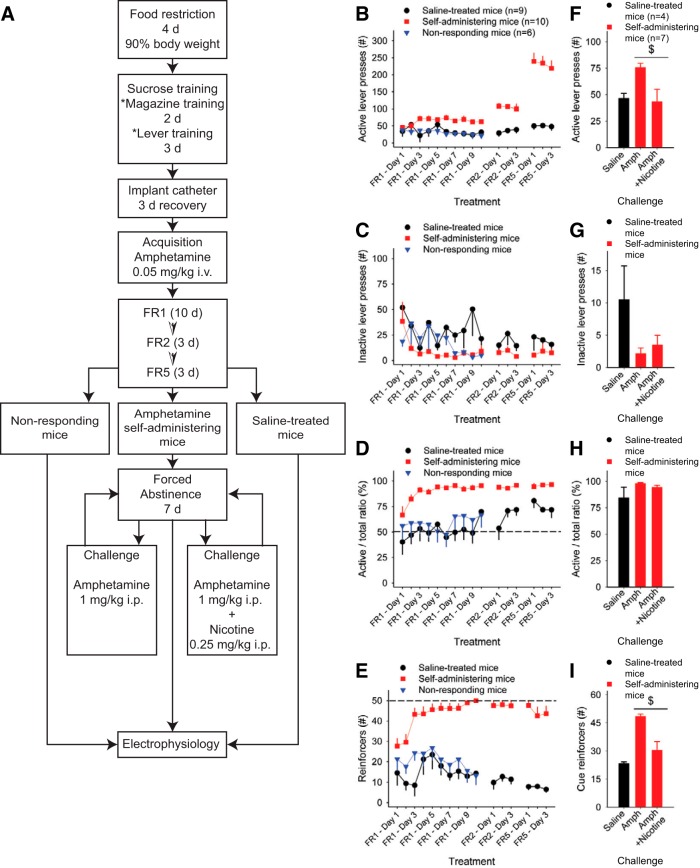
Mice self-administer amphetamine. ***A***, Acquisition of amphetamine self-administration: Following sucrose pretraining, mice were trained to self-administer amphetamine under increasing schedules of reinforcement. After 10 d on FR1, mice that had not achieved stable responding (<20 reinforcers during the last 3 d of FR1) were designated as nonresponding mice and were excluded from the remainder of the behavioral study. Thereafter the saline-treated mice and amphetamine self-administering mice underwent a further 6 d on FR2 and FR5 schedules of reinforcement. Following this acquisition phase, the amphetamine self-administering mice underwent 7 d of abstinence. This was followed by an amphetamine challenge in the same operant boxes and protocol as used during intravenous self-administration to assess the incubation of drug seeking behaviors. The effect of nicotine on this amphetamine challenge was also assessed. ***B***, In comparison with saline-treated mice, amphetamine self-administering mice increased the number of active lever presses. ***C***, The number of inactive lever presses was similar across all groups. ***D***, Compared with saline-treated mice, the amphetamine self-administering mice showed drug-lever association, as assessed by the ratio of active to total (inactive + active) lever presses (%). ***E***, During FR1, FR2, and FR5 schedules of reinforcement, amphetamine self-administering mice achieved a greater number of reinforcers than mice treated with saline, often reaching 50, the maximum allowed during the 2 h session. ***F***, Following forced abstinence, nicotine reduced the total number of active lever presses observed after an amphetamine challenge (***G***) without altering the inactive lever presses. ***H***, There was no effect of nicotine on lever discrimination, as shown by the ratio of active/total lever presses. ***I***, The administration of nicotine with amphetamine reduced the number of reward-associated cues. The effect of a saline injection on saline-treated mice is shown for comparison with amphetamine self-administering mice. For all panels, ^$^*p*<0.05, paired *t* test.

Although amphetamine self-administration has been demonstrated in rats, there are no published examples of mice acquiring amphetamine self-administration. We used a multistage protocol to obtain amphetamine self-administration through an indwelling jugular catheter (Fig. 1*A*). After a period of habituation and sucrose exposure, we trained mice to obtain a 20% sucrose reward, delivered to the magazine, upon pressing either of two levers for 3 consecutive days (Table 8). We then implanted an intravenous catheter in a jugular vein, and after 3 d of recovery, began amphetamine self-administration (0.05 mg/kg/infusion per reinforcer) using fixed, increasing schedules of reinforcement. Within the group of 20 mice self-administering amphetamine with patent catheters, 10 mice obtained 50 reinforcers, the maximum number allowed, after 10 d at FR1, during which one lever press resulted in one reinforcer. We designated this group as amphetamine self-administering mice. Of the 10 remaining mice, five were removed due to occluded catheters. Five additional mice that had not obtained more than an average of 20 reinforcers during the last three sessions of FR1 were designated as nonresponding mice and excluded from the rest of the behavioral study. The 10 amphetamine self-administering mice progressed from FR1 to FR2 and FR5 schedules of reinforcement. A further 10 saline-treated mice underwent the identical procedure, but received saline instead of amphetamine. Saline-treated mice were included in all parts of the acquisition phase, and 5 of 10 underwent abstinence and a saline challenge. Figure 1*B*–*E* demonstrates the different measures recorded during the acquisition phase.

**Table 8.** Sucrose pre-training

	Day 1	Day 2	Day 3
Left Lever	21±3	20±3	27±5
Right Lever	43±6	37±4	41±5*
Time	40±5	28±3	20±1^##^

The lever preference (left or right) and time taken to obtain 30 reinforcers during 3 consecutive days of sucrose pretraining. *p<0.05 versus day 3, left lever, ^##^*p*<0.01 versus time to obtain 30 reinforcers on day 1.

The number of active lever presses by the amphetamine self-administering mice increased from 45 to 76 during the first 3 d of FR1 and then plateaued at 60–70 presses for the remaining 7 d of FR1 (Fig. 1*B*). Thereafter, the number of active lever presses increased to ∼120 at FR2 and to ∼240 at FR5. This increase in the number of active presses required to receive the same number of infusions demonstrates the motivation of this group to receive amphetamine.

The active and inactive lever pressing of saline-treated and nonresponding mice was minimal (Fig. 1*C*). Likewise, amphetamine self-administering mice did not often press the inactive lever, but their active lever presses increased with the fixed ratio of reinforcers (saline-treated vs amphetamine self-administering active lever presses over time; *F*_(15,120)_=26.55, *p*<0.0001^a^, two-way ANOVA; Fig. 1*B*). The percentage ratio of active to total (active + inactive) lever presses assessed the ability to discriminate the effect of pressing the active from inactive lever. The amphetamine self-administering mice achieved an average of 90±3, 95±1, and 95±1% active/total lever presses during FR1, FR2, and FR5, respectively (Fig. 1*D*). This was greater than the active/total lever ratio in saline-treated mice (*F*_(1,7)_=149, *p*<0.0001^b^, two-way ANOVA) who achieved 55±3, 65±6, and 75±3% during FR1, FR2, and FR5. The amphetamine self-administering mice obtained a greater number of drug reinforcers than saline-treated mice (*F*_(15,120)_=3.837, *p*<0.0001^c^, two-way ANOVA) and consistently reached 50, the maximum allowed, during the 2 h session of each schedule (Fig. 1*E*).

We assessed the effect of abstinence on drug-seeking behavior. After 7 d of forced abstinence, we injected the amphetamine self-administering mice with either amphetamine alone (1 mg/kg, i.p.) or with amphetamine (1 mg/kg, i.p.) and nicotine (0.25 mg/kg, i.p.). We then placed the mice in the same operant chamber as used during the acquisition phase for 30 min. This amphetamine challenge resulted in a slight incubation effect when compared with the first 30 min period of the final FR5 session (76±4 vs 63±4 active lever presses during the incubation vs FR5 session, respectively; *p*=0.01^d^, *t*=3.7, df=6, paired *t* test). Nicotine coadministration with amphetamine reduced both the number of active lever presses (−44±14%; *p*=0.02^e^, *t*=3.04, df=5, paired *t* test) and the number of reinforcing cues obtained (−37±9%; *p*=0.02^f^, *t*=3.5, df=5, paired *t* test; Fig. 1*F*–*I*), resulting in a similar profile of drug-seeking responses as seen in saline-treated mice challenged with a saline injection. Nicotine treatment did not alter lever discrimination, as neither the number of inactive lever presses (*p*=0.07^g^, *t*=2.2, df=6, paired *t* test) nor the percentage ratio of active/total lever presses changed (*p*=0.15^h^, *t*=1.7, df=5, paired *t* test; Fig. 1*G*,*H*).

### The tonic firing of ChIs is lower in amphetamine self-administering mice

**Figure 2.**
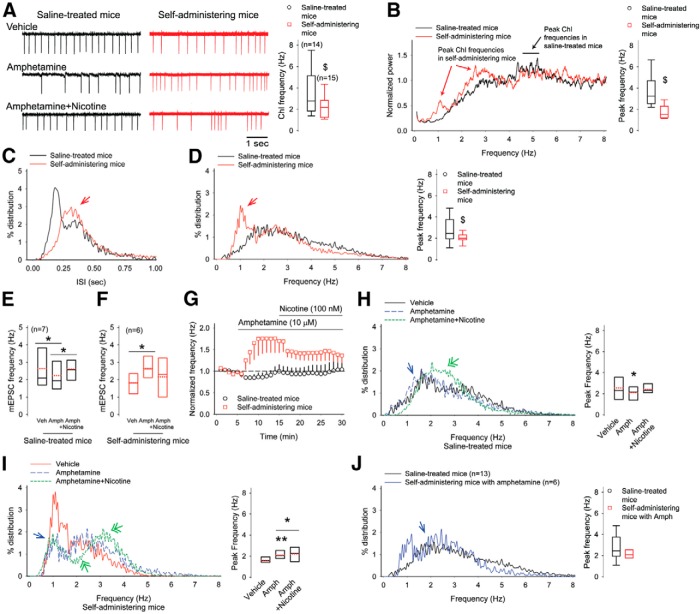
Amphetamine self-administration modifies ChI firing. ***A***, Representative traces of cell-attached recordings in ChIs from saline-treated and amphetamine self-administering mice (left). The average baseline firing frequency (right) was lower in ChIs from amphetamine self-administering mice; This is shown in box-and-whisker plots where the median is shown as a solid line, the mean value is dotted red, the ends of the box indicate the 25^th^ and 75th percentiles, and the bars indicate the 10^th^ and 90^th^ percentiles. For all panels, ^$^*p*<0.05, Student’s *t* test; *p<0.05 and **p<0.01, paired *t* test. ***B***, The normalized power distribution (left) and the average peak frequency (right) show prominent low-frequency activity in ChIs from amphetamine self-administering mice. ***C***, The ISI distribution for ChIs from saline-treated and amphetamine self-administering mice (arrow). ***D***, Frequency distributions (left) and their average peaks (right) show the prominent low-frequency distribution (1/ISI) of ChI firing from amphetamine self-administering mice (arrow). ***E***, Mean±SE frequencies of ChIs from saline-treated and (***F***) amphetamine self-administering mice in response to amphetamine (Amph) or amphetamine with nicotine. ***G***, Normalized firing frequencies over time for the experiments shown in ***E*** and ***F***. ***H***, The frequency distribution (left) and the average peak frequency (right) of ChIs from saline-treated mice. Amphetamine produced a small increase in activity at 0.5 Hz (arrow). Nicotine blocked the inhibition caused by amphetamine and increased activity at 2.5 Hz (double arrow). ***I***, Frequency distribution (left) and the average peak frequency (right) of ChIs from amphetamine self-administering mice show that amphetamine reduced 1–2 Hz activity (arrow). The addition of nicotine moderated the potentiating effect of amphetamine by reducing activity between 2 and 3 Hz, while increasing 3–4 Hz activity (double arrows). ***J***, When exposed to amphetamine, the bimodal frequency distribution (left) and average peak frequency (right) of ChIs from amphetamine self-administering mice converged with the unimodal frequency distribution of ChIs from saline-treated mice.

Psychostimulants enhance DA availability and induce long-lasting plasticity in ChI activity and ACh availability (Bickerdike and Abercrombie, 1997, 1999; Bamford et al., 2008; Witten et al., 2010; Wang et al., 2013a), yet their effect on ChI firing is poorly understood. To determine whether amphetamine self-administration modifies ChI activity *ex vivo*, we measured their spontaneous firing using cell-attached recordings in acute striatal slices from amphetamine self-administering and saline-treated mice. The baseline firing frequency of ChIs from amphetamine self-administering mice (2.25±0.03 Hz; range 1.05–4.5 Hz; *n*=15 cells) was 37% lower than ChIs from saline-treated mice (3.58±0.58 Hz; range 1.05–9.06 Hz; *n*=14 cells; *p*=0.03^i^, Student’s *t* test; Fig. 2*A*).

Through their widespread dendritic and axonal fields, ChIs modulate corticostriatal excitability through changes in the frequency of their tonic firing (Wilson et al., 1990). We developed computer algorithms to examine the power and relative distribution of individual firing frequencies in ChIs from saline-treated and amphetamine self-administering mice (Tables 1–6). The Welch’s power spectral density estimate of ChI activity from saline-treated mice revealed a unimodal distribution, with a single local maximum of 3.68±0.4 Hz centered on the average frequency of 3.58±0.58 Hz (Fig. 2*B*). Most (∼90%) individual ChIs from amphetamine self-administering mice also demonstrated a unimodal frequency distribution, but their averaged contributions appeared bimodal, with the uppermost peak frequency centered on the average frequency of ChIs from saline-treated mice, whereas the second maxima was centered on lower frequencies ∼1 Hz (Fig. 2*B*–*D*). The peak frequencies identified by the power spectral density estimate approximated the frequency distributions derived from their ISIs and showed that ChI spiking in amphetamine self-administering mice was lower than saline-treated mice due to an increased distribution of low-frequency activity between 0.5 and 1.5 Hz (Fig. 2*C*,*D*).

### An amphetamine challenge increases tonic ChI firing in amphetamine self-administering mice

To determine whether an amphetamine challenge modified ChIs activity *ex vivo*, amphetamine (10 µM) was bath-applied in a concentration that elevates striatal DA concentrations to ∼3 μM (Bamford et al., 2004b), via a reversal of the DA transporter (Sulzer, 2011). Amphetamine reduced the firing frequency of ChIs from saline-treated mice by 15±4% (2.64±0.53 Hz in vehicle vs 2.24±0.28 Hz with amphetamine; *n*=7 cells; *p*=0.006^j^, paired *t* test), but increased the firing rate of ChIs from amphetamine self-administering mice by 58±34% (1.82±0.27 Hz vs 2.65±0.39 Hz with amphetamine; *n*=6 cells; *p*=0.04^k^, paired *t* test; Fig. 2E-G). Spectral analysis showed that amphetamine reduced the average peak frequency in ChIs from saline-treated mice by augmenting low-frequency activity between 0.5 Hz and 1.5 Hz (Fig. 2H). In ChIs from amphetamine self-administering mice, an amphetamine challenge increased activity by diminishing the contribution of those same frequencies (Fig. 2I). Treatment with amphetamine did not reorganize the bimodal frequency distribution of ChI activity in amphetamine self-administering mice, but modified the skewed, fat-tailed firing frequency to better approximate the spectra of ChIs from saline-treated mice (Fig. 2E, F, and J).

### Nicotine opposes the change in tonic ChI firing caused by an amphetamine challenge

Nicotine attenuated amphetamine-induced drug-seeking behaviors (Fig. 1), and we determined whether nicotine (100 nM) *ex vivo*, in a concentration that approximates serum levels in smokers (Dani and Harris, 2005), modifies the effect of an amphetamine challenge on tonic ChI firing. When administered with amphetamine *in vitro*, nicotine blocked amphetamine inhibition of ChI activity in saline-treated mice (2.64±0.53 Hz in vehicle vs 2.61±0.31 Hz with amphetamine and nicotine; *p*=0.3^p^, paired *t* test) by enhancing frequencies between 2 and 3 Hz (Fig. 2*E*–*H*). Conversely, in ChIs from amphetamine self-administering mice, nicotine suppressed amphetamine-mediated excitation (1.82±0.27 Hz in vehicle vs 2.16±0.51 Hz with amphetamine and nicotine; *p*=0.3^q^, paired *t* test) by diminishing these same frequencies (Fig. 2*E*–*G*,*I*). Nicotine modified higher frequencies and blocked the change in tonic firing caused by an amphetamine challenge.

### Amphetamine self-administration reduces burst firing in ChIs

**Figure 3.**
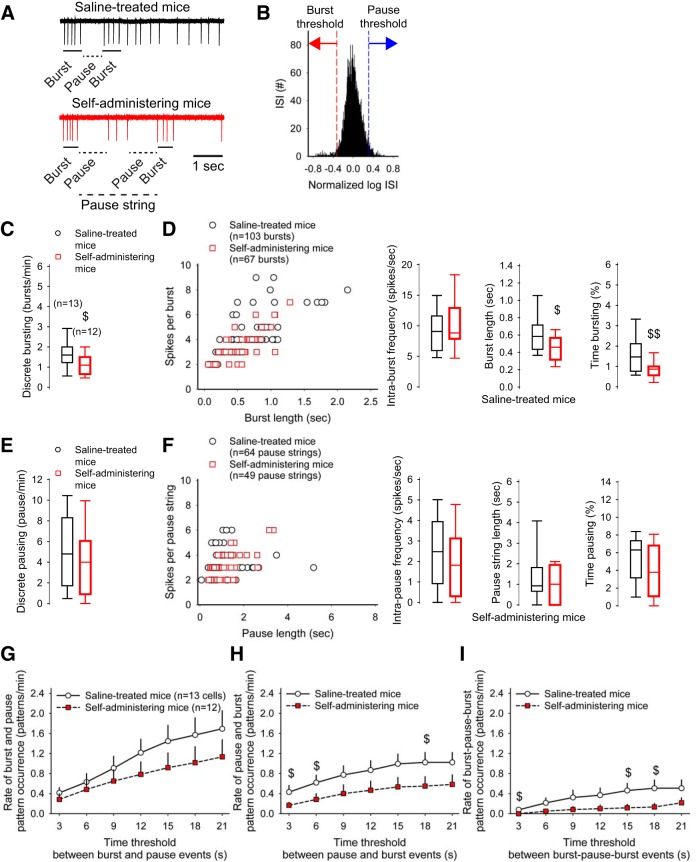
Amphetamine self-administration alters burst firing. ***A***, The representative traces from a cell-attached recording of ChIs from saline-treated and amphetamine self-administering mice show burst, pause, and pause-string activity. The pause response of ChIs begins with an initial depolarizing phase followed by a pause in spike firing and ensuing rebound excitation. In this example, the ChI from a self-administering mouse lacks rebound excitation. ***B***, The representative normalized log_10_ ISI distribution demonstrates the RGS method for determining burst and pause thresholds for each ChI. The log_10_ ISI values of burst (−0.324) and pause thresholds (0.311) for this cell correspond to the top 0.5 percentile and bottom 0.5 percentile. ***C***, The average frequency (bursts/min) of discrete bursts was lower in ChIs from amphetamine self-administering mice. For all panels, $*p*<0.05, $$*p*<0.01, Student’s t test. ***D***, The histogram (left) compares the length of each burst with the frequency of spikes contained within that burst. The average intraburst frequency (spikes/s), average burst length (s), and the average percentage of time spent bursting determines the cell’s burstiness. Compared to saline-treated mice, ChIs from amphetamine self-administering mice had a similar intraburst frequency, because the bursts contained a lower number of spikes and were of shorter duration. ***E***, The average frequency (pauses/min) of discrete pause activity. ***F***, The histogram (left) compares the length of each pause string with the frequency of spikes contained within that pause string. The average intrapause string frequency (spikes/s), average pause string length (s), and the average percentage of time used by pause strings was similar in ChIs from saline-treated and amphetamine self-administering mice. ***G***, The minimum time thresholds that connect burst and pause events are compared with rate of burst-pause, (***H***) pause-burst, and (***I***) burst-pause-burst occurrence.

Reward-reporting stimuli increase the activity of dopaminergic neurons and correlate with a change in ChI activity throughout the striatum (Aosaki et al., 1994). These conditioned responses modify the tonic activity of ChIs by producing burst, pause, and rebound burst-firing patterns of various lengths that modify downstream network activity (Graybiel et al., 1994; Fig. 3*A*). Thus, minute, as well as volume-transmitted changes in ACh availability at nicotinic receptors may encourage gaiting at corticostriatal synapses. Electrophysiological studies have shown that although tonic activity may remain stable, changes in the burst and pause activity of DA neurons may play a role in degenerative disease (Lobb, 2014; Lobb and Jaeger, 2015) and could be altered by psychostimulant exposure. Indeed, ChIs show distinct region-specific responses to cocaine (Benhamou et al., 2014), but alterations in ChI burst or pause activity that accompany amphetamine self-administration are unclear.

We used the RGS method to detect and quantify how amphetamine self-administration modifies unique burst and pause patterns in individual ChIs. The RGS method has been successful in identifying changes in bursting patterns of DA neurons (Branch et al., 2013; Lobb and Jaeger, 2015), as it exhibits a robust adaptability to varying firing rates through two steps: normalization of local ISI distributions and iterative addition of normalized ISIs, based on the p value obtained from global Gaussian statistics of the whole spike train, to burst and pause strings. (Ko et al., 2012). RGS thresholds for burst and pause activity were determined in ChIs from saline-treated (*n*=13 cells) and amphetamine self-administering mice (*n*=12 cells) using Gaussian probability distribution of the ISIs for each cell (see Materials and Methods; Fig. 3*B*). To determine whether amphetamine self-administration modified the cycle and extent of burst activity, we expanded our burst-firing analysis to measure any change in the burstiness of the cell, defined by its intraburst frequency, burst length, and the percentage of time spent bursting. We also measured discrete pauses (single pauses in activity without intervening spikes), pause strings (contiguous discrete pauses with intermittent low-frequency spikes), intrapause frequency, pause length, and the percentage of time spent pausing.

Compared to saline-treated mice, ChIs from amphetamine self-administering mice had a lower frequency of discrete bursts (1.65±0.19 bursts/min vs 1.12±0.15 bursts/min for amphetamine self-administering mice; *p*=0.03^r^, Student’s *t* test) and a reduction in their burstiness; the average burst length (612±63 ms vs 447±42 ms for amphetamine self-administering mice; *p*=0.02^s^, Student’s *t* test) and the percentage of time spent bursting (1.57±0.26% vs 0.83±0.13% for amphetamine self-administering mice; *p*=0.01^t^, Student’s *t* test) decreased, whereas the intraburst frequency remained constant (8.99±0.1 spikes/s vs 10.1±0.13 spikes/s for amphetamine self-administering mice; Fig. 3*C*,*D*). There was no change in discrete pauses or pause strings (Fig. 3*E*,*F*). Thus, amphetamine self-administration reduced both tonic firing and bursting and uncoupled burst and pause activity in ChIs (Fig. 3*G*–*I*).

### Amphetamine and nicotine modify burst and pause activity in ChIs

**Figure 4.**
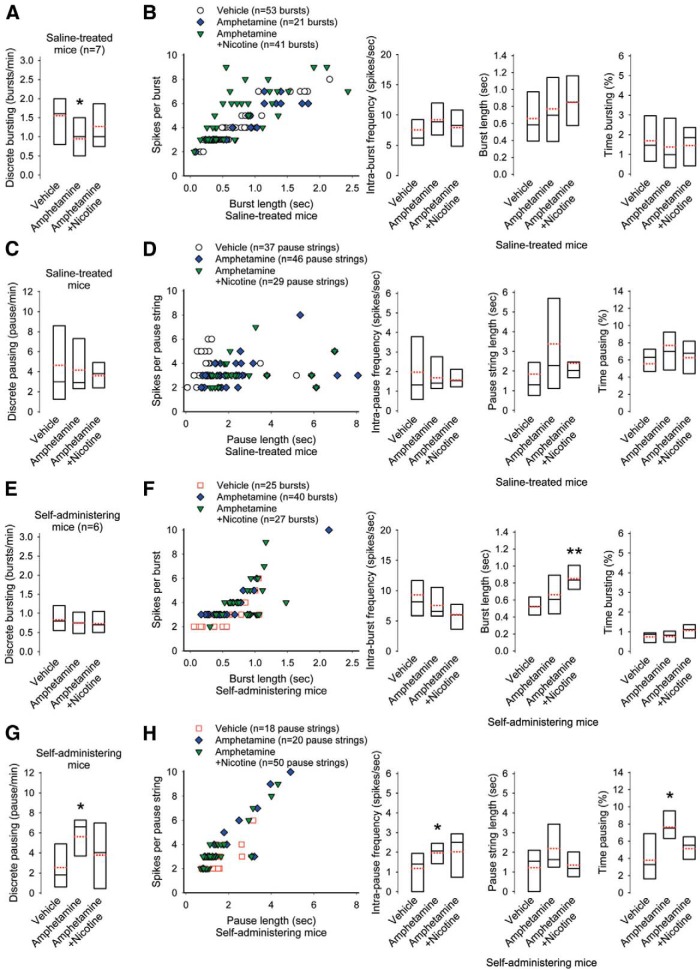
Nicotine and amphetamine modify burst and pause activity. ***A***, Nicotine blocked the reduction of discrete burst activity by amphetamine in ChIs from saline-treated mice. For all panels, *p<0.05, **p<0.01, paired *t* test. ***B***, The burstiness of ChIs from saline-treated mice was unaffected by amphetamine or the coadministration of nicotine. ***C***, Amphetamine with nicotine or without did not change discrete pausing or (***D***) pause strings in ChIs from saline-treated mice. ***E***, In ChIs from amphetamine self-administering mice, amphetamine or coadministered nicotine did not modify discrete bursting (***F***) but nicotine increased the length of bursting. ***G***, Nicotine blocked the increase in discrete pausing by amphetamine in ChIs from amphetamine self-administering mice. ***H***, In ChIs from amphetamine self-administering mice, an amphetamine challenge produced a nicotine-dependent enhancement of pause strings by boosting the intra-pause frequency and the percentage of time spent pausing.

Nicotine opposes the change in tonic ChI firing caused by an amphetamine challenge. We therefore determined if a nicotine challenge would modify the burst and pause activity in ChIs from saline-treated and amphetamine self-administering mice. In ChIs from saline-treated mice (*n*=7 cells), amphetamine *in vitro* reduced the frequency of discrete bursts (1.55±0.31 vs 0.94±0.17 bursts/min without or with amphetamine; *p*=0.02^u^, paired *t* test), but did not modify pausing (Fig. 4*A*–*D*). When combined with amphetamine, nicotine blocked the reduction in discrete bursts (1.55±0.31 bursts/min vs 1.26±0.28 bursts/min with amphetamine and nicotine) and had no effect on pausing.

In ChIs from amphetamine self-administering mice, an amphetamine challenge *in vitro* had no effect on burst activity. Instead, amphetamine increased the frequency of discrete pauses (2.53±0.99 pauses/min vs 5.62±1.02 pauses/min with amphetamine; *p*=0.04^v^, paired *t* test) and enhanced the pause strings by increasing the intra-pause frequency (1.19±0.42 spikes/s vs 1.96±0.27 spikes/s with amphetamine; *p*=0.03^w^, paired *t* test), as well as the percentage of time spent pausing (3.79±1.14% vs 7.54±1.02% with amphetamine; *p*=0.04^x^, paired *t* test; Fig. 4*G*,*H*). When added to amphetamine, nicotine increased the burst length and blocked the rise in discrete pausing and pause strings. Therefore, an amphetamine challenge reduced tonic firing and bursting in ChIs from saline-treated mice and reversed the depression in the ChI activity of amphetamine self-administering mice by increasing tonic firing. In summary, nicotine attenuated drug-seeking behaviors and opposed the changes in ChI activity that occurred following an amphetamine challenge.

### Nicotine reduces tonic, burst, and pause activity of ChIs

**Figure 5.**
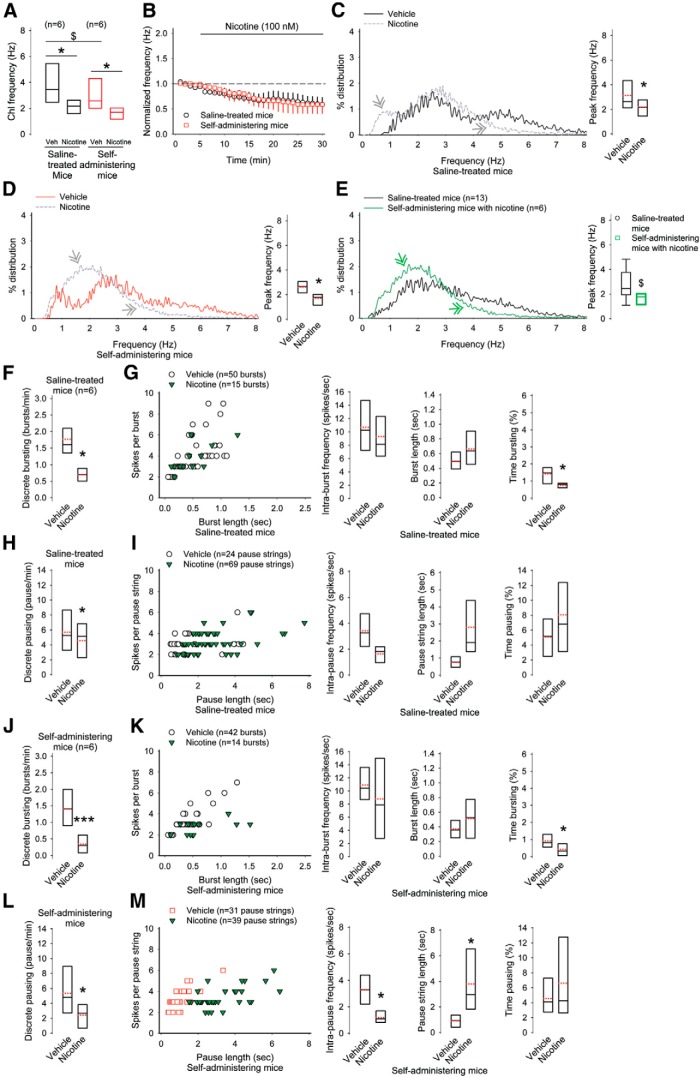
Nicotine inhibits ChI activity. ***A***, Nicotine reduced the mean frequencies of ChIs from saline-treated and amphetamine self-administering mice. For all panels, ^$^*p*<0.05, Student’s *t* test and *p<0.05, ***p<0.001, paired *t* test. ***B***, Normalized-firing frequencies over time for the experiments shown in ***A***. ***C***, In ChIs from saline-treated mice, the spectra (left) show that nicotine (double arrows) increased low frequencies, whereas reducing higher frequencies and the average peak frequency (right). Nicotine shifted the unipolar distribution of ChIs from saline-treated mice toward the bimodal distribution of average frequencies in amphetamine self-administering mice. ***D***, In ChIs from amphetamine self-administering mice, nicotine increased low frequencies, reduced high frequencies, and suppressed the average peak frequency. ***E***, Nicotine shifted the bimodal distribution of average frequencies in amphetamine self-administering mice toward the unipolar distribution of ChIs from saline-treated mice. ***F***, In ChIs from saline-treated mice, nicotine suppressed discrete bursting, (***G***) the percentage of time spent bursting, and (***H***) discrete pausing, but (***I***) did not alter pause strings. ***J***, In ChIs from amphetamine self-administering mice, nicotine suppressed discrete bursting, (***K***) the percentage of time spent bursting, and (***L***) discrete pausing. **M**, Amphetamine self-administration modified pause strings; the intra-pause spike frequency decreased whereas the length of the pause string increased.

Next, we examined nicotine’s modulation of ChI activity in the absence of amphetamine. Bath-applied nicotine, at a concentration that desensitizes high-affinity α4β2*-type nicotinic receptors (Lester and Dani, 1995; Wooltorton et al., 2003), decreased the firing frequency of ChIs from both saline-treated mice (−41±8%; 3.76±0.67 Hz vs 2.08±0.26 Hz with nicotine; *n*=6 cells; *p*=0.03^y^, paired *t* test) and amphetamine self-administering mice (−39±18%; 2.88±0.52 Hz vs 1.57±0.22 Hz with nicotine; *n*=6 cells; *p*=0.04^z^, paired *t* test; Fig. 5*A*,*B*). Nicotine reduced tonic firing by increasing low-frequency activity and by reducing higher frequencies (Fig. 5*C*,*D*). Nicotine converted the spectra of ChIs from amphetamine self-administering mice to a unimodal distribution that was characteristic of ChIs from saline-treated mice (Fig. 5*E*).

Nicotine *in vitro* reduced the frequency of discrete bursts (1.77±0.26 bursts/min vs 0.7±0.09 bursts/min with nicotine; *p*=0.01^aa^, paired *t* test; Fig. 5*F*) and the burstiness of ChIs from saline-treated mice. The intraburst frequency and burst length remained constant, whereas the percentage of time spent bursting decreased (1.42±0.22% vs 0.73±0.09% with nicotine; *p*=0.01^ab^, paired *t* test; Fig. 5*G*). Nicotine preserved the coupling between burst and pause activity (Aosaki et al., 1994), as the reduction in bursting was accompanied by a drop in discrete pauses (5.73±1.33 pauses/min vs 4.59±1.06 pauses/min with nicotine; *p*=0.04^ac^, paired *t* test; Fig. 5*H*,*I*).

Similar to ChIs from saline-treated mice, nicotine *in vitro* reduced the frequency of discrete bursts (1.4±0.23 bursts/min vs 0.34±0.12 bursts/min with nicotine; *p*=0.0008^ad^, paired *t* test; Fig. 5*J*) and the burstiness of ChIs from amphetamine self-administering mice. The interburst frequency and burst length remained constant, whereas the percentage of time spent bursting decreased (0.92±0.23% vs 0.42±0.19% with nicotine; *p*=0.01^ae^, paired *t* test; Fig. 5*K*). The reduction in bursts accompanied a decrease in discrete pauses (5.37±1.52 spikes/s vs 2.42±0.72 spikes/s with nicotine; *p*=0.02^af^, paired *t* test; Fig. 5*L*) and an increase in pause strings: The intra-pause frequency decreased (3.3±058 pauses/min vs 1.19±0.19 pauses/min with nicotine; *p*=0.02^ag^, paired *t* test), whereas the pause string length increased (0.92±0.26 s vs 3.8±0.92 s with nicotine; *p*=0.02^ah^, paired *t* test). There was no change in percentage of time spent pausing, suggesting that the discrete pauses coalesced into pause strings with reduced intra-pause activity (Fig. 5*M*). Thus, nicotine reduced tonic, burst, and pause activity in both amphetamine naive and self-administering mice, perhaps by its interactions with α4β2*- or α7*-type nicotinic autoreceptors located on ChIs (Azam et al., 2003).

### Amphetamine self-administration promotes a CPD in corticostriatal activity

**Figure 6.**
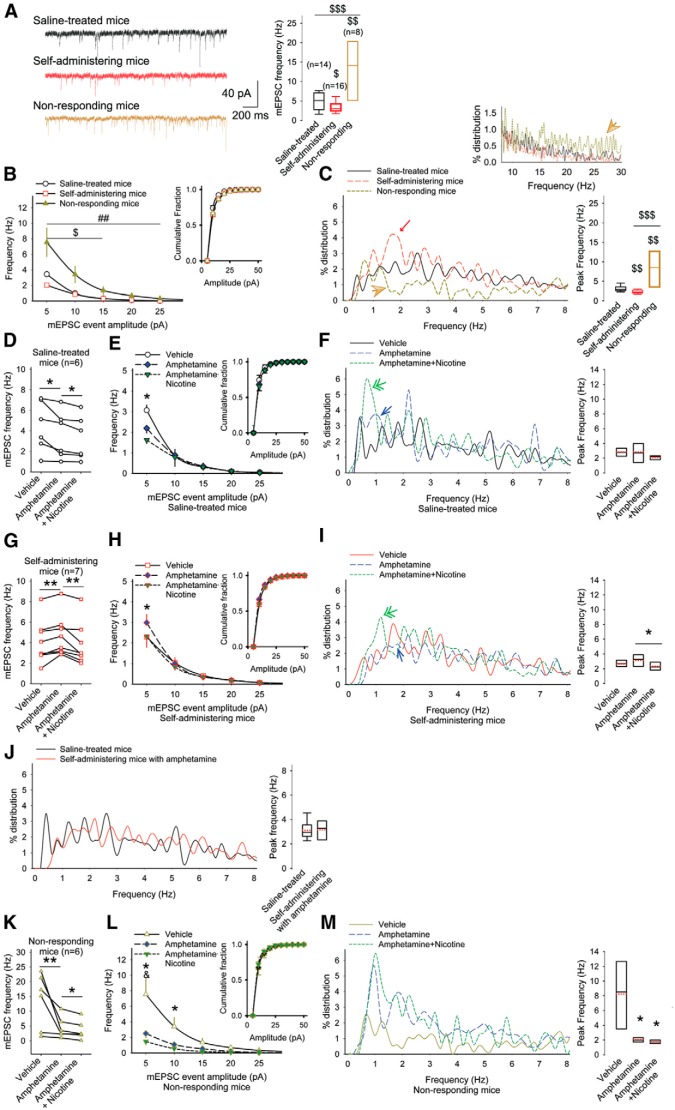
Nicotine blocks PPP. ***A***, Representative traces (left) and plots (right) show that the frequency of mEPSC in MSNs from saline-treated mice was greater than amphetamine self-administering mice but far less than those in nonresponding mice. ^$^*p*<0.05, ^$$^*p*<0.01, ^$$$^*p*<0.001, Student’s *t* test. ***B***, Compared with saline-treated mice, amphetamine self-administration reduced the frequency of low-amplitude mEPSCs, while the frequency of mEPSCs was broadly higher in nonresponding mice. ^$^*p*<0.05, saline-treated versus amphetamine self-administering mice; ^##^*p*<0.01, saline-treated versus nonresponding mice; Bonferroni *t* test. Inset, The cumulative mEPSC amplitude distribution was unchanged across groups. ***C***, Frequency distributions (left) and the average peak frequency (right) show a prominent low-frequency distribution of mEPSCs from amphetamine self-administering mice (small arrow) and more broadly distributed firing frequencies in MSNs from nonresponding mice (arrowhead; inset). ^$$^*p*<0.01, ^$$$^*p*<0.001, Student’s *t* test. ***D***, Responses of individual MSNs from saline-treated mice show that amphetamine, and the addition of nicotine, reduced the frequency of mEPSCs. *p<0.05, paired *t* test. ***E***, Amphetamine with nicotine or without decreased the frequency of low-amplitude mEPSCs. *p<0.05, Bonferroni *t* test, amphetamine compared with either vehicle or amphetamine with nicotine. Inset, The mEPSC amplitude distribution was unchanged. ***F***, Frequency distributions (left) and the average peak frequency (right) of mEPSCs from saline-treated mice shows that both amphetamine (arrow) and amphetamine with nicotine (double arrow) increased low-frequency events. ***G***, In MSNs from amphetamine self-administering mice, nicotine blocked the increase in mEPSCs that followed amphetamine. *p<0.05, paired *t* test. ***H***, Amphetamine augmented the frequency of low-amplitude mEPSCs, but had no effect on their amplitude distribution (inset). *p<0.05, Bonferroni *t* test, amphetamine compared with either saline or amphetamine with nicotine. ***I***, The distribution (left) and the average peak frequency (right) of mEPSCs from amphetamine self-administering mice show that amphetamine reduces 1–2 Hz activity (arrow). The addition of nicotine moderated the effect of amphetamine by increasing 1–2 Hz activity (double arrows). *p<0.05, paired *t* test. ***J***, The distribution (left) and average peak frequency (right) show that mEPSCs s from saline-treated mice were similar to those from amphetamine self-administering mice exposed to amphetamine. ***K***, Both amphetamine and amphetamine with nicotine reduced the mEPSC frequency in MSNs from nonresponding mice. *p<0.05, **p<0.01, paired *t* test. ***L***, Amphetamine and nicotine reduced low-amplitude mEPSCs, but had no effect on their amplitude distribution (inset). *p<0.05, amphetamine compared with either vehicle or amphetamine with nicotine; ^&^*p*<0.01, vehicle compared with either amphetamine or amphetamine with nicotine; Bonferroni *t* test. ***M***, The frequency distribution (left) and average peak frequency (right) of mEPSCs from nonresponding mice show that amphetamine with (double arrow) or without nicotine (arrow) increases low-frequency events. *p<0.05, paired *t* test.

ChIs modulate corticostriatal activity through pre- and postsynaptic muscarinic receptors (Calabresi et al., 2000; Witten et al., 2010), but their control over corticostriatal activity derived through presynaptic nicotinic receptors remains unclear. We used whole-cell recordings in acute striatal slices to measure mEPSCs in MSNs from saline-treated, amphetamine self-administering, and nonresponding mice. The baseline frequency of mEPSCs in MSNs from amphetamine self-administering mice (3.45±0.42 Hz) was lower than mEPSCs in MSNs from saline-treated mice (4.92±0.59 Hz; *p*=0.04^ai^, Student’s *t* test; Fig. 6*A*), consistent with a depression in corticostriatal activity. The baseline frequency of mEPSCs in MSNs from nonresponding mice (13.59±3.82 Hz) was much higher than saline-treated (*p*=0.003^aj^, Student’s *t* test) or amphetamine self-administering mice (*p*=0.0003^ak^, Student’s *t* test). Differences in mEPSCs between these groups of mice mainly affected low-amplitude currents and there were no differences in the mEPSC amplitude distribution (Fig. 6*B*), suggesting that corticostriatal plasticity was presynaptic (Van der Kloot, 1991). The depression of mEPSC in MSNs from amphetamine self-administering mice was secondary to an increase in low-frequency activity distributed between 0.5 and 2 Hz, whereas the prominence of mEPSCs in MSNs from nonresponding mice was due to the persistence of frequencies >7 Hz (Fig. 6*C*).

### An amphetamine challenge promotes PPP in self-administering mice

Having established that amphetamine self-administration depresses corticostriatal activity, we then determined how an amphetamine (10 μm) challenge *in vitro* would modify this plasticity. In saline-treated mice, amphetamine reduced mEPSC frequency by 20±8% (4.42±1.1 Hz in vehicle vs 3.58±1.03 Hz with amphetamine; *n*=8 cells; *p*=0.04^al^, paired *t* test; Fig. 6*D*), consistent with D2R-mediated depression of corticostriatal activity (Flores-Hernández et al., 1997; Bamford et al., 2004b). In the presence of amphetamine, nicotine (100 nm) further reduced the frequency of mEPSCs (−8±2%; 3.28±0.96 Hz in amphetamine and nicotine; *p*=0.04^am^, paired *t* test compared with amphetamine alone; *p*=0.01^an^, compared with vehicle, paired *t* test). Both amphetamine and amphetamine with nicotine *in vitro* reduced low-amplitude currents, but not the mEPSC amplitude distribution (Fig. 6*E*). The frequency spectra showed that both amphetamine and amphetamine with nicotine reduced mEPSC frequencies by increasing low-frequency activity between 0.5 and 1.5 Hz (Fig. 6*F*).

In amphetamine self-administering mice, amphetamine *in vitro* paradoxically increased mEPSC frequency by 21±5% (4.09±0.8 Hz vs 4.64±0.8 Hz with amphetamine; *n*=8 cells; *p*=0.004^ao^, paired *t* test; Fig. 6*G*), thereby enhancing mEPSC frequency to the same level seen in MSNs from saline-treated mice without amphetamine (4.42±1.1 Hz). Nicotine *in vitro* blocked amphetamine-generated PPP as it reduced mEPSC frequency in amphetamine self-administering mice (−20±5%; 3.8±0.8 Hz in amphetamine and nicotine; *p*=0.006^ap^, compared with amphetamine alone, paired *t* test) by depressing low-amplitude currents (Fig. 6*G*,*H*). Amphetamine *in vitro* increased the mEPSC frequency by reducing low-frequency events, whereas nicotine *in vitro* blocked PPP by enhancing these same frequencies (Fig. 6*I*). Similar to responses in ChIs (Fig. 2*J*), treatment with amphetamine stabilized mEPSCs in amphetamine self-administering mice, as their event frequencies approximated mEPSCs from saline-treated mice (Fig. 6*J*).

The baseline mEPSC frequency in MSNs from nonresponding mice was much higher than that from either amphetamine naive mice or self-administering mice. Amphetamine *in vitro* significantly depressed mEPSCs by −54±13% (13.6±4.18 Hz in vehicle vs 4.41±1.62 Hz in amphetamine; *n*=6 cells; *p*=0.003^aq^, paired *t* test; Fig. 6*K*). When combined with amphetamine, nicotine *in vitro* produced a further reduction in mEPSCs (−31±8%; 2.41±0.92 in amphetamine and nicotine; *p*=0.02^ar^, compared with amphetamine alone, paired *t* test). Amphetamine and nicotine *in vitro* reduced mEPSC frequency by reducing small amplitude currents and amplifying low-frequency events (Fig. 6*L*,*M*).

### Nicotine modulates corticostriatal activity through α4β2*- and α7*-type nicotinic receptors

**Figure 7.**
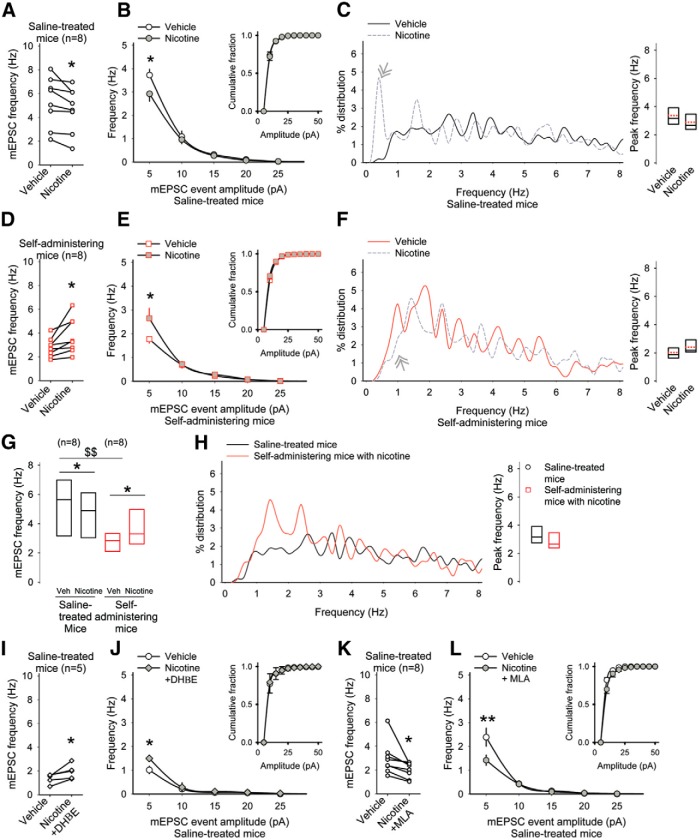
Nicotine *in vitro* blocks CPD through α7*- and α4β2*-type nicotinic receptors. ***A***, Responses of individual MSNs show that nicotine inhibited corticostriatal activity in saline-treated mice by (***B***) reducing high-frequency, low-amplitude mEPSCs, but had no effect on their amplitude distribution (inset). For ***B***, ***E***, ***J***, *p<0.05, Bonferroni *t* test; for all other panels, *p<0.05, paired t test and ^$$^*p*<0.01, Student’s t test. ***C***, The frequency distribution (left) and the average peak frequency (right) of mEPSCs. Nicotine increased low-frequency mEPSCs in saline-treated mice. ***D***, Nicotine increased the frequency of mEPSCs in MSNs from amphetamine self-administering mice by (***E***) augmenting high-frequency, low-amplitude mEPSCs, while having no effect on their amplitude distribution (inset). ***F***, The frequency distribution (left) and average peak frequency (right) of mEPSCs. Nicotine reduced low-frequency mEPSCs in amphetamine self-administering mice. ***G***, Mean±SE frequencies of mEPSCs from saline-treated and amphetamine self-administering mice in response to nicotine. ***H***, The frequency distribution (left) and average peak frequencies (right) compares mEPSCs in MSNs from saline-treated mice with those from amphetamine self-administering mice exposed to nicotine *in vitro*. ***I***, The α4β2*-type receptor antagonist DHβE blocked the synaptic depression generated by nicotine and (***J***) increased high-frequency, low-amplitude synaptic events. ***K***, The α7*-type nicotinic receptor antagonist MLA reduced (***L***) high-frequency, low-amplitude mEPSCs. ***J***, ***L***, Insets show similar mEPSC amplitude distributions.

We determined whether the parallel alterations in ChI spiking and corticostriatal activity that followed amphetamine self-administration were promoted through α7*- as well as α4β2*-type nicotinic receptors acting at glutamatergic synapses in the dorsal striatum (McGehee et al., 1995; Marchi et al., 2002; Pakkanen et al., 2005). Nicotine *in vitro* reduced the frequency of mEPSCs in MSNs from saline-treated mice by 13±5% (5.29±0.8 Hz vs 4.67±0.71 Hz with nicotine; *n*=8 cells; *p*=0.04^as^, paired *t* test), primarily by decreasing small amplitude currents and by magnifying low-frequency events (Fig. 7*A*–*C*). In comparison, nicotine *in vitro* increased the frequency of mEPSCs in MSNs from amphetamine self-administering mice (33±7%; 2.81±0.3 Hz vs 3.4±0.56 Hz with nicotine; *n*=8 cells; *p*=0.03^at^, paired *t* test) by modulating these same currents and frequencies (Fig. 7*D*–*F*). Nicotine did not alter the cumulative amplitude distribution of mEPSC in MSNs from saline-treated or amphetamine self-administering mice, suggesting modulation at presynaptic sites (Fig. 7*B*,*E*, inset). Amphetamine self-administration reduced the baseline frequency of mEPSCs in MSNs (5.29±0.8 Hz for saline-treated vs 2.81±0.3 Hz for amphetamine self-administering mice; *p*=0.007^au^, Student’s *t* test) and nicotine blocked this depression by increasing the frequency of mEPSCs (4.42±1.1 Hz; *p*=0.2^av^, compared with mEPSCs from saline-treated mice, Student’s *t* test; Fig. 7*G*). Therefore, nicotine *in vitro* blocked CPD and PPP in amphetamine self-administering mice and helped stabilize the frequency distribution of mEPSCs in these cells (Fig. 7*H*).

We used saline-treated mice to test whether nicotine modulation at corticostriatal terminals acts through α4β2*- and α7*-type nicotinic receptors. The mEPSC frequency increased by 46±18% when the α4β2*-type receptor antagonist dihydro-beta-erythroidine (DHβE; 40 nM) was added to nicotine (1.37±0.21 Hz vs 1.93±0.31 Hz with nicotine and DHβE; *n*=5 cells; *p*=0.04^aw^, paired *t* test; Fig. 7*I*,*J*), consistent with α4β2*-type receptor desensitization at this concentration of nicotine (Lester and Dani, 1995; Wooltorton et al., 2003). When the α7*-type nicotinic receptor antagonist methyllycaconitine (MLA; 40 nM) was applied with nicotine, the mEPSC frequency was inhibited (−29±6%; 2.98±0.54 Hz vs 2±0.24 Hz with nicotine and MCT; *n*=8 cells; *p*=0.04^ax^, paired *t* test; Fig. 7*K*,*L*), beyond that seen with nicotine alone (−13±5%; *p*=0.04, Student’s *t* test). This suggests that nicotine can boost corticostriatal activity through low-affinity α7*-type nicotinic receptors, which desensitize with much higher levels of nicotine (>500 nm; Lester and Dani, 1995; Wooltorton et al., 2003). These data show that nicotine can block CPD at corticostriatal terminals and that alterations in ChI activity and ACh availability following repeated amphetamine likely modify corticostriatal activity through α7*-type, as well as α4β2*-type nicotinic receptors.

### Nicotine modifies behaviors and corticostriatal activity in noncontingent amphetamine-treated mice

Behavioral sensitization characterizes the progressive and enduring enhancement found in many amphetamine-induced behaviors (Robinson and Camp, 1987). More specifically, locomotor sensitization depends on psychostimulant-induced plasticity of ACh-releasing ChIs in the dorsal striatum that promote long-lasting changes in corticostriatal activity (Bamford et al., 2008; Witten et al., 2010; Wang et al., 2013a). In contrast to noncontingent amphetamine-treated mice, mice self-administering amphetamine (contingent amphetamine-administration) had a 57% larger reduction in baseline ChI activity in withdrawal (24% vs 37% for amphetamine self-administering mice) but a 34% smaller increase in activity following an amphetamine challenge *in vitro* (88±27% vs 57±34% for amphetamine self-administering mice; data not shown in figures). Similarly, the baseline frequency of mEPSC in MSNs was depressed following amphetamine self-administration, but was unchanged following noncontingent treatment. Further, an amphetamine challenge *in vitro* produced a much smaller increase in mEPSC frequency in amphetamine self-administering mice (107±21% vs 21±11% for amphetamine self-administering mice; data not shown in figures). Therefore, the contingent administration of amphetamine produced a greater depression in ChI firing and corticostriatal activity, but a lower rebound following an amphetamine challenge.

### Nicotine modifies the incubation of locomotor sensitization and corticostriatal activity

**Figure 8.**
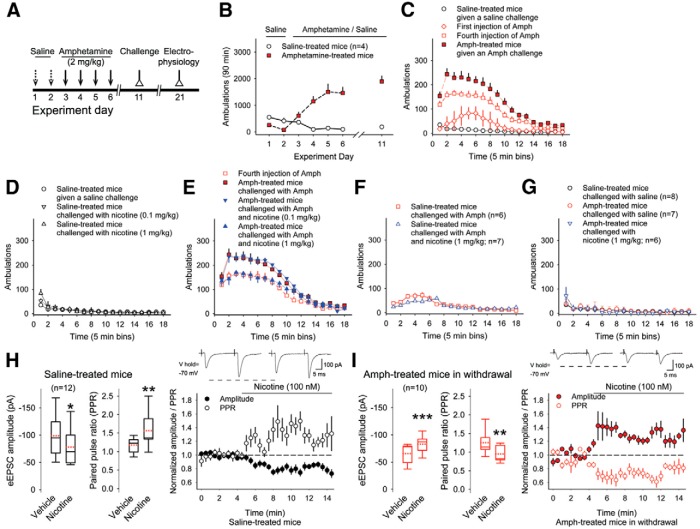
Nicotine modifies locomotor sensitization and corticostriatal activity. ***A***, Protocol for testing the effect of amphetamine and nicotine on locomotor sensitization. The amphetamine treatment group received saline injections for the first 2 d, whereas the saline treatment group received saline on all days. ***B***, Average locomotor ambulations over 90 min in mice treated with either saline or amphetamine. ***C***, Interval locomotor ambulations over 90 min; compare the ambulations following the first injection of amphetamine with those following an amphetamine challenge in withdrawal. Also shown are ambulations in saline-treated mice following a saline challenge. ***D***, Interval locomotor ambulations over 90 min in saline-treated mice that follow an injection of low-dose nicotine, high-dose nicotine, or saline. ***E***, Interval locomotor ambulations over 90 min in amphetamine-treated mice. Ambulations that follow the first injection of amphetamine are compared with ambulations that follow an amphetamine challenge in withdrawal, with and without simultaneous treatment with nicotine *in vivo*. Low-dose nicotine had little effect on ambulations, whereas the higher dose of nicotine reduced ambulations to those observed during the last daily treatment of amphetamine. ***F***, Interval locomotor ambulations over 90 min in saline-treated mice following an amphetamine challenge, with and without high-dose nicotine. ***G***, Interval locomotor ambulations over 90 min in amphetamine-treated mice following a challenge with saline or high-dose nicotine are compared to ambulations in saline-treated mice challenged with saline. ***H***, In MSNs from saline-treated mice, nicotine *in vitro* decreased the amplitude of the first eEPSC and increased the PPR. For all panels, *p<0.05, **p<0.01, ***p<0.001, paired *t* test. ***I***, In MSNs from amphetamine-treated mice, nicotine increased the amplitude of the first eEPSC and decreased the PPR.

To assess whether nicotine might alter the expression of locomotor sensitization, as well as incubation of drug-seeking behaviors, we treated mice with repeated amphetamine and challenged them with amphetamine and nicotinic receptor ligands in withdrawal. Mice received saline for 2 d and amphetamine (2 mg/kg/d, i.p.) for 4 d. Following a 5 d withdrawal, we challenged mice with amphetamine (2 mg/kg, i.p.; Fig. *8A*). Mice increased their locomotor ambulations following each amphetamine treatment [*F*_(5,18)_=11.6, *p*<0.001^ay^, repeated-measures (rm)-ANOVA; Fig. 8*B*]. Compared to locomotor ambulations following their first (*F*_(17,102)_=5.19, *p*<0.001^az^, two-way rm-ANOVA) and fourth day of amphetamine treatment (*F*_(17,102)_=3.71, *p*<0.001^az^, two-way rm-ANOVA), ambulations increased when mice were challenged with amphetamine in withdrawal (Fig. 8*C*).

To test whether nicotine modified locomotor ambulations in withdrawal, mice received either low- (0.1 mg/kg, i.p.) or high- dose (1 mg/kg, i.p.) nicotine on withdrawal day 5. Neither dose of nicotine had an effect on locomotion in saline-treated mice (*p*=0.9^ba^, two-way, rm-ANOVA; Fig. 8*D*). In amphetamine-treated mice challenged with amphetamine, low-dose nicotine had no effect on locomotor ambulations (*F*_(17,102)_=0.1, *p*=1^bb^, two-way rm-ANOVA), whereas the higher dose of nicotine reduced locomotor ambulations (*F*_(17,102)_=3.19, *p*=0.01^bc^, two-way rm-ANOVA; Fig. 8*E*). Ambulations following high-dose nicotine were similar to those following the fourth dose of amphetamine, indicating that nicotine suppressed the expression of locomotor sensitization. Locomotor testing following an amphetamine challenge in nonsensitized, saline-exposed mice showed that nicotine (1 mg/kg) did not block the acute stimulating effect of amphetamine on locomotion (*p*=0.9^bd^, two-way rm-ANOVA; Fig. 8*F*). These same saline and nicotine (1 mg/kg) challenges in amphetamine-treated mice revealed similar ambulations compared with saline-treated mice challenged with saline (*p*=0.9^be^, two-way rm-ANOVA; Fig. 8*G*). This indicates that amphetamine-experienced mice lack conditioned locomotor activity elicited by placement into the amphetamine-associated chamber and show that nicotine did not modify locomotor activity in the absence of amphetamine.

It was previously shown that withdrawal from repeated injections of amphetamine produced CPD and PPP following reinstatement (Wang et al., 2013a). In another group of similarly treated mice, we performed electrophysiological recordings to determine whether nicotine *in vitro* modifies corticostriatal activity in withdrawal. eEPSCs in MSNs were obtained in response to 50 ms paired-pulses delivered to the motor cortex. In MSNs from saline-treated mice, puff-applied nicotine decreased the amplitude of the first current of the pair by 19±5% (−99±11 pA vs −78±10 pA with nicotine; *n*=12 cells; *p*=0.02^bf^, paired *t* test) and increased the PPR by 34±12% (1.18±0.06 vs 1.56±0.15 with nicotine; *p*=0.009^bg^, paired *t* test; Fig. 8*H*), indicating a reduction in corticostriatal excitation. In MSNs from amphetamine-treated mice, nicotine increased the amplitude of the first current by 30±6% (−66±6 pA vs −83±5 pA with nicotine; *n*=10 cells; *p*<0.001^bh^, paired *t* test) and decreased the PPR by 23±3% (1.25±0.11 vs 0.95±0.07 with nicotine; *p*=0.013^bi^, paired *t* test; Fig. 8*I*). Compared with MSNs from saline-treated mice (*n*=12 cells), the amplitude of the first pulse of the pair was lower in MSNs from amphetamine-treated mice in withdrawal (*n*=10 cells; −99±11 pA for saline-treated vs −66±6 pA in withdrawal; *p*=0.02^bj^, Student’s *t* test; Fig. 8, compare H, I). By increasing the amplitude of the first eEPSC, nicotine blocked the synaptic depression in withdrawal (−99±11 pA for saline-treated in vehicle vs −83±5 pA for withdrawal with nicotine; *p*=0.2^bk^, Student’s *t* test). Therefore, alterations in ACh availability extend to models of noncontingent amphetamine-treated mice because nicotine suppressed the expression of locomotor sensitization and although nicotine *in vitro* reduced eEPSCs in saline-treated mice, it blocked synaptic depression in withdrawal by enhancing corticostriatal activity.

## Discussion

The transition from psychostimulant use to dependence involves changes in corticostriatal activity in consequence of intermittent DA release (Kalivas, 2009; Sulzer, 2011). Repeated exposure to amphetamine, which releases DA (Bamford et al., 2004b), promotes long-lasting and parallel changes in drug-primed rewarding, locomotor sensitization, ChI firing, and corticostriatal activity. This suggests that ChIs in the striatum participate in addictive behaviors and contribute to normal motor and cognitive function by promoting allostasis and sensitized responses (Ahmed and Koob, 2005). Our data show that the rewarding properties of amphetamine and the potentiation of locomotor sensitization are dependent on ACh because nicotine attenuated both responding to an amphetamine challenge after abstinence and sensitized locomotor responses to an amphetamine challenge in withdrawal.

**Figure 9.**
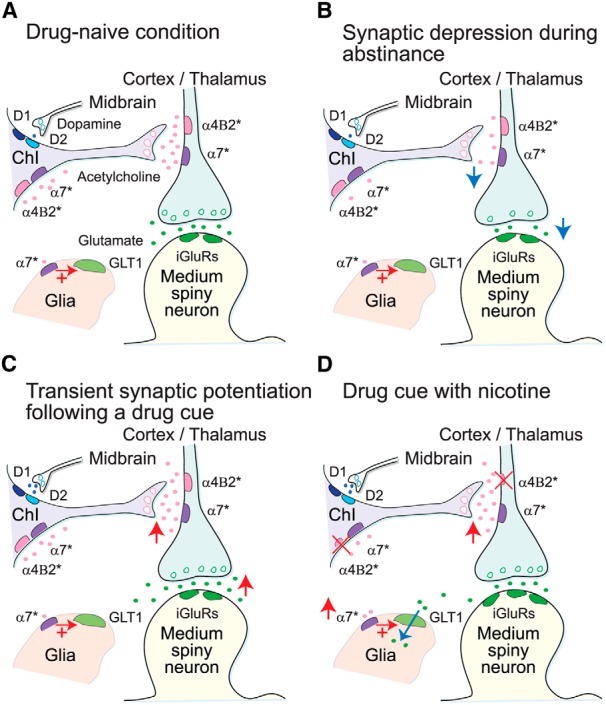
Proposed mechanism for nicotinic modulation of striatal synaptic plasticity following amphetamine self-administration. ***A***, The simplified striatal circuit is composed of MSNs, ChIs, and glia. Glutamate released from cortical and thalamic afferents excites MSNs through ionotropic glutamate receptors (iGluRs). In the drug naive condition, ACh efflux from ChIs binds to excitatory α4β2*- and α7*-type nicotinic receptors and modulates ChI firing and glutamate release from corticostriatal terminals (Bamford et al., 2008; Wang et al., 2013a). The glial glutamate transporter (GLT1) limits the access of synaptic glutamate to the extracellular space (Kalivas, 2009). ***B***, Synaptic depression develops during abstinence from repeated amphetamine. Repeated DA release by amphetamine stimulates inhibitory D2-class receptors on ChIs (Yan et al., 1997; Wang et al., 2013a). The reduction in tonic excitation at α4β2*- and α7*-type nicotinic receptors depresses corticostriatal activity. Low tonic levels of ACh are supported during drug withdrawal by nicotinic autoreceptors that lower the set point of ChI firing (Wang et al., 2013a). ***C***, A drug cue promotes transient synaptic potentiation. Because of the lowered set point of ChI activity, DA released by a drug cue stimulates ACh efflux via excitatory D1-class receptors on ChIs (Yan and Surmeier, 1997; Wang et al., 2013a). The increase in ACh boosts glutamate release, causing PPP. ***D***, Nicotine prevents PPP. The concentration of nicotine achieved in smokers (100 nm) desensitizes excitatory α4β2*-type nicotinic receptors (Dani and Harris, 2005). The lack of nicotinic receptor excitation reduces ChI spiking and corticostriatal activity in the drug naive condition and during abstinence. The drug cue enhances ACh tone but desensitization prevents PPP in response to drug reinstatement. Activation of α7*-type nicotinic receptors expressed on astrocytes (Pirttimaki et al., 2013) recruit AMPA receptors to glutamatergic terminals (Wang et al., 2013b), have a protective effect on oxidative stress (Liu et al., 2015), and increase activity of GLT1 to clear glutamate from the extracellular space (Kalivas, 2009).

Preclinical and clinical evidence suggests that cigarette smoking and nicotinic receptors participate in psychostimulant addiction (Rahman, 2013; Mello et al., 2014; Verrico et al., 2014). Contingent models of drug-seeking behavior in rodents have shown an interaction between nicotine and methamphetamine incubation (Shepard et al., 2004; Hiranita et al., 2006; Neugebauer et al., 2010; Pipkin et al., 2014). However, this interaction is complex and altered by diverse factors such as age, dose, drug pairing, and frequency of use. No studies have investigated the effect of acute nicotine on amphetamine incubation in mice. Using a standard self-administration protocol based on fixed schedules of reinforcement, we demonstrate that mice acquire amphetamine self-administration behaviors and develop the motivation to obtain this drug. As this experiment is technically challenging, we then used a within-subject design (Ma et al., 2013), to assess the effect of nicotine on amphetamine-seeking behaviors following abstinence. When combined with the electrophysiological analysis of MSNs and ChIs, amphetamine self-administration provides a valuable tool to explore the striatal circuit- and ChI-adaptations induced by contingent drug exposure (Fig. 9).

ChIs in the dorsal striatum respond to conditioned sensory stimuli that signal reward delivery and elicit behavioral reactions (Aosaki et al., 1994). Measures of ChI frequencies *ex vivo* revealed a reduction in tonic ChI activity from amphetamine self-administering mice. Although having no effect on pausing, an amphetamine challenge following abstinence decreased the frequency of discrete bursts and burstiness, thereby dissociating or uncoupling burst activity from pausing in the absence of DA-releasing stimuli. Nicotine reduced tonic firing, discrete bursts, burstiness, and discrete pauses in ChIs from both saline-treated and amphetamine self-administering mice. When nicotine suppressed already low tonic firing in amphetamine self-administering mice, the discrete pauses coalesced into pause strings. As ChIs are widely distributed throughout the striatum, the uncoupling of burst and pause activity could serve to modulate the activity of surrounding neurons and mediate rewarding behaviors.

The literature on where these forms of plasticity and their related behaviors occur is unclear. However, much evidence indicates that the circuitry underlying amphetamine-induced locomotion is mediated by NAc, whereas stereotypies are mediated by dorsal striatum (Asher and Aghajanian, 1974; Pijnenburg et al., 1976; Segal, 1976; Castall et al., 1977). Furthermore, the development of sensitization is associated with prefrontal cortex and VTA, whereas expression is associated with the prefrontal cortex and the NAc (McFarland and Kalivas, 2001; Kalivas, 2009). The data presented here suggests that the potentiation of locomotor sensitization is also associated with long-term plasticity in the dorsal striatum. The role of ChIs in the dorsal striatum has been associated with cue-dependent behaviors and the expression of locomotor responses to repeated amphetamine (Wang et al., 2013a), thus agreeing with the results of our self-administration studies. Repeated psychostimulant use produces similar forms of glutamatergic plasticity in the ventral (Kalivas, 2009) and dorsal striatum (Bamford et al., 2008), suggesting that both structures play a role in the behavioral potentiation seen in locomotor sensitization and self-administration.

Psychostimulant-released DA reduces ChI firing through D2-class DA receptors on ChIs (Yan et al., 1997), and the firing rate of ChIs remained suppressed following amphetamine self-administration. Data support the hypothesis that a lasting inhibition of ChI activity in withdrawal occurs through a reduction in tonic excitation at autoregulatory receptors, because nicotine reduced spontaneous firing and concentered the spectral signatures in ChIs from both saline-treated and amphetamine self-administering mice. Therefore, a decrease in tonic excitation at nicotinic autoreceptors on ChIs (Azam et al., 2003) in amphetamine self-administering mice may maintain the depression of ChI firing in withdrawal.

The differential action of excitatory D1- and inhibitory D2-class DA receptors located on ChIs establish a set point for ChI activity (Abercrombie and DeBoer, 1997; Wang et al., 2013a). An amphetamine challenge reduced tonic firing and discrete bursting in ChIs from amphetamine naive mice, but increased the already low tonic firing in ChIs from amphetamine self-administering mice. Amphetamine regulates ChI activity through DA receptors on ChIs, where the reduction in tonic firing in saline-treated mice occurs through D2 receptors, whereas the excitation during amphetamine withdrawal are generated through heightened responses mediated through D1 receptors (Wang et al., 2013a). Thus, repeated DA release during acquisition followed by abstinence may create a shift in the activity set point of ChIs and reverse their response to an amphetamine challenge or to cues paired with the drug. Nicotine suppressed these two divergent DA-dependent changes in ChI activity, suggesting that nicotinic autoreceptors participate in DA-dependent plasticity. Although amphetamine modulated ChI firing at frequencies centered on 1 Hz, nicotine shifted ChI activity toward a pre-existing spectral baseline by modulating frequencies centered on 3 Hz. Nicotine may oppose DA-dependent changes in ChI activity by stabilizing membrane potentials required for GABA receptor modulation of ChIs (DeBoer and Westerink, 1994) or hyperpolarization-activated cation channels that encode their autonomous firing (Bennett et al., 2000).

Data indicate that ChI firing (and ACh availability) promotes parallel changes in corticostriatal activity. Similar to responses in ChIs, amphetamine *in vitro* reduced corticostriatal activity in saline-treated mice and repeated amphetamine promoted CPD in withdrawal. Nicotine blocked both CPD and amphetamine-induced PPP, signifying that nicotinic receptors are both necessary and sufficient to promote long-lasting corticostriatal plasticity. PPP was absent in nonresponding mice even though they were exposed to small doses of amphetamine *in vivo*. Although the responses to amphetamine and nicotine were similar to saline-treated mice, nonresponding mice had a much higher frequency of mEPSCs at baseline and consequently, amphetamine produced a robust depression in activity. Future investigations of both the behavioral responding to different doses of amphetamine and cellular adaptations will be required to understand the basis of these observations.

Nicotine reduced amphetamine-seeking behaviors and the expression of locomotor sensitization. Although nicotinic receptors are located throughout the brain, nicotine may modify ChI and corticostriatal activity through α4β2*- and α7*-type nicotinic receptors that are located on ChIs (Azam et al., 2003) and presynaptic corticostriatal terminals (Marchi et al., 2002; Pakkanen et al., 2005). The level of nicotine (100 nm) has been shown to desensitize α4β2*-type nicotinic receptors (Lester and Dani, 1995; Wooltorton et al., 2003) and may therefore act to lessen ChI excitation and corticostriatal activity. In saline-treated mice, nicotine reduced corticostriatal excitation caused by synaptic ACh, likely by desensitizing and internalizing excitatory α4β2* receptors (Zhou et al., 2002; Pakkanen et al., 2005). In amphetamine self-administering mice, the decrease in ACh availability may promote CPD by reducing excitation at both α4β2*- and α7*-type nicotinic receptors, whereas the increase in ChI activity following an amphetamine challenge supports PPP. Nicotine may therefore supplement the low availability of ACh to increase corticostriatal activity, but desensitizes the α4β2*-type nicotinic receptor and prevents PPP when given in combination with amphetamine. Thus, nicotine may offset corticostriatal plasticity by preventing the change in ACh availability that occurs during the incubation of drug-seeking behavior. However, the modulation of ChI and corticostriatal activity by ACh and nicotine is likely complex and involves a host of nicotine receptor subtypes with different sensitivities and levels of expression.

Increasing evidence suggests that ChI firing and ACh availability promote an imbalance in glutamate release from corticostriatal terminals (Bamford et al., 2008; Wang et al., 2013a) to produce motor and neuropsychological symptoms of drug dependence (Robinson and Camp, 1987; Kalivas, 2009). Although psychostimulant-releasing DA reduces ChI and corticostriatal activity in amphetamine naive mice, the increase in DA and ACh availability following a drug challenge in sensitized mice promotes a PPP of corticostriatal activity that correlates with the increase in locomotor activity and promotes sensitization by specifically activating D1R-expressing MSNs (Gass and Olive, 2008; Wang et al., 2013a). Together, CPD and PPP provide a mechanism by which a drug challenge promotes locomotor sensitization and allostasis (Ahmed and Koob, 2005). The much higher basal level of corticostriatal activity in nonresponding mice may provide some protection against PPP, because an amphetamine challenge reduced the frequency of mEPSCs to that found in saline-treated mice.

These data extend to observations of cocaine-induced glutamatergic neuroadaptations, where drug withdrawal and reinstatement accompany a reversible reduction in extracellular (Kalivas, 2009) and synaptic glutamate (Kozell and Meshul, 2003). Sensitization models of incentive salience (Robinson and Camp, 1987) suggest that a supra-physiological glutamatergic drive promotes compulsive drug-seeking in addicts by decreasing the value of natural rewards (Kalivas, 2009). While demonstrating that CPD and PPP play key roles in sensitization, these experiments show that alterations in ACh may overlap with the plasticity and learning associated with self-administration and incubation. Thus, DA modulation of ACh release may provide a set point for glutamate availability (Ahmed and Koob, 2005) that extends to the escalation of drug intake (Hansen and Mark, 2007).

ChIs appear to support motor coordination, cognitive flexibility, and locomotion through ACh interactions with striatal projection neurons (Bamford et al., 2008; Witten et al., 2010; Wang et al., 2013a). Because DA provokes state changes in corticostriatal activity, our findings may explain how salient experiences encode behaviors and automatic movements. Changes in ACh and glutamate availability are promoted by repeated DA release. Our findings are consistent with human trials, which demonstrate that nicotinic receptors and changes in glutamate transmission participate in the pathogenesis of many neuropsychological disorders (Mihailescu and Drucker-Colín , 2000; Gass and Olive, 2008), including Parkinson’s disease where nicotinic receptor ligands prevent the disruption of striatal and cholinergic activity following DA depletion (Quik, 2004). Similarly, therapeutic approaches using nicotinic receptor ligands that prevent PPP may facilitate the extinction of drug seeking behavior. If provided during the process of sensitization, these drugs would prevent CPD and disrupt the reinforcing effects of drugs in addicts (Gass and Olive, 2008; Reissner and Kalivas, 2010). Together, these observations suggest the need for additional circuit-level and behavioral experiments in conjunction with epidemiological studies to determine whether pharmacological targeting of nicotinic receptors can modify the development of drug seeking and reduce relapse.

## Synthesis

The decision was a result of the Reviewing Editor Christian Hansel and the peer reviewers coming together and discussing their recommendations until a consensus was reached. A fact-based synthesis statement explaining their decision and outlining what is needed to prepare a revision is listed below. The following reviewer(s) agreed to reveal their identity: Daniel McGehee

This is the first mouse amphetamine self administration study and does a careful job of decoding the synaptic changes involved in drug self-administration. It is a promising paper that may strongly contribute to understanding the physiology of addiction and is a technical tour-de-force. In its current stage, though, the manuscript lacks clarity, because it is not well justified why specific measures where done, and why specific metrics have been highlighted in the analysis. The manuscript would greatly benefit from better structuring and clarity.

This manuscript outlines a series of technically challenging experiments linking changes in the physiology of striatal cholinergic interneurons (ChI) to amphetamine self-administration (SA). The authors also test the impact of nicotine exposure on ChI physiology. First, the authors establish a mouse model of amphetamine self-aministration (SA), which is an important contribution to the field. They exploit that model to conduct an exhaustive investigation of ChI firing rate/burst activity and synaptic inputs. They then put the mice through a 7day AMPH withdrawal paradigm and test the impact of AMPH challenge with and without nicotine exposure. However, the manuscript suffers from poor writing, with little or no justification for nearly every metric.

1) The authors appear to be unaware of the dramatic differences in the plasticity effects of amphetamine versus cocaine shown by Marina Wolf's laboratory (see Nelson et al 2009). This data may help justify exploration of AMPH-induced changes in ChI physiology

2) There is no clear justification for examining the physiology of ChI's in the dorsal striatum. The experimental model needs to be explained in the introduction and then the motivation for each experiment should be mentioned briefly in the results section.

3) The various documented firing rate parameters are, in many cases, redundant and unnecessary. Do we really need to see burst frequency &amp; duration as well as pause frequency &amp; duration? If so, the reason for these apparently overlapping measures needs to be justified. Discussion of inter-burst intervals, inter-pause intervals that merely restate the effects on frequency are confusing and not needed. The authors need to explain why each metric is important and focus on the changes that are relevant to the drug induced changes.

4) The mechanisms underlying the observed changes in firing rate and synaptic transmission are not explored adequately. How much of the effect of AMPH is dopamine receptor-dependent? These studies are not practical for the chronic in vitro tests, but the reviewers suggest to provide more mechanistic investigation for the acute, in vitro measures by using DA / Ach antagonists. The reviewers agree that this is not a requirement for acceptance of the manuscript, but would make the paper stronger and higher impact.

This manuscript reports for the first time (to the authors and our knowledge) the acquisition and analysis of self-administration for amphetamine in mice. Furthermore, the authors developed various macros (power spectra, frequency spectra, and Robust Gaussian Surprise method) for performing an "in depth" analysis of spike data. These macros allow analysis of how changes in specific frequency ranges produce effects at different stages of the amphetamine exposure protocols, and detects burst, pauses, and the parameters associated to these firing patterns in cholinergic interneurons. They also provided language for creating these macros in MatLab. Thus, this manuscript provides new tools for studying addiction mechanisms and firing patterns.

The authors address whether nicotinic signaling modulation is a possible target for minimizing long-term changes associated to amphetamine addiction, as has been previously shown for cocaine addiction. The authors found that pairing nicotine with amphetamine during withdrawal abolishes self-administration and the potentiation observed in locomotion, but does not abolish sensitization per se. From the cellular point of view (ex vivo experiments), nicotine counteracts amphetamine effect on cholinergic interneurons and glutamatergic synapses on medium spiny neurons. These results provide evidence supporting a role for nicotinic receptors in modulating the striatal circuit during withdrawal.

The experiments for this manuscript are well performed. However, there is one major discussion point to address, and the authors must verify some minor points and typos.

Discussion Point:

The literature on where these forms of plasticity and related behaviors occur are confusing but important to this paper.

The authors atribute locomotor sensitization to long-term changes in the dorsal striatum, and the literature is congruent on that point.

In contrast, however, much evidence indicates that amphetamine-induced locomotion is mediated by nucleus accumbens, while stereotypies are mediated by dorsal striatum (Brain Research, 82 (1974) 1-12, Brain Research, 123 (I977) 89-111, Brain Research, 94 (1975) 507-522, Eur. J. Pharmacol., 35 (1976) 45-58, Physiol. Psychol., 12 (1984) 159-162). Furthermore, development of sensitization is associated with prefrontal cortex and VTA, while expression is associated to nAcc and PFC.

The role of cholinergic interneurons in the dorsal striatum has been associated to cue-dependent behaviors, thus agreeing with the self-administration behaviors.

The authors should compare the similar effects ("behavioral potentiation") they are observing for both locomotor sensitization and self-administration, and discuss how their proposed mechamism might occur in both structures (dorsal and nAcc).

Minor Points:

1. Page 5, paragraph A-Acquisition: "We tested catheter patency by infusing 1% propofol (20 μl) after FR1 and again after FR3 and FR."

FR = FR5?

2. Page 14, paragraph 2: "The amphetamine self-administering mice achieved an average of 90{plus minus}3, 95{plus minus}1, and 95{plus minus}1% active/total lever presses during FR1, FR2, and FR5, respectively (Fig. 1D)."

The figure 1D use active/inactive. Which one is the correct?

3. Page 22, paragraph 2: "Tonic firing of ChIs modulates corticostriatal activity through

pre-and postsynaptic muscarinic receptor"

pre- and

4. please run a careful spell check, including on figures ("abstenance")
